# Systematic evaluation and meta-analysis of transcardiac intracavitary and transesophageal echocardiography-guided left atrial appendage occlusion surgery

**DOI:** 10.3389/fcvm.2026.1701359

**Published:** 2026-03-03

**Authors:** Bing Luo, Yizhen Niu, Liwei Zhang, Zhihua Cheng

**Affiliations:** 1 The First Affiliated Hospital of Hebei North University, Zhangjiakou, Hebei, China; 2 Department of Ultrasound, The First Affiliated Hospital of Hebei North University, Zhangjiakou, Hebei, China; 3 Hebei North University, Zhangjiakou, China

**Keywords:** effectiveness, esophageal echocardiography, intracardiac ultrasound, LAAO, meta-analysis, safety

## Abstract

**Objective:**

To systematically evaluate the differences in safety, efficacy, and economics between intracardiac ultrasound (ICE) and transesophageal echocardiography (TEE)-guided LAAO (LAAO), and to provide an evidence-based rationale for the selection of clinical image-guided modalities.

**Methods:**

PubMed, Embase, Cochrane Library, Web of Science and Wanfang databases were searched to include randomized controlled trials and observational studies comparing ICE with TEE-guided LAAO, strictly following PRISMA guidelines. Two investigators independently screened the literature, extracted data and assessed the risk of bias (ROBINS-I and Cochrane tools). Meta-analysis was performed using RevMan 5.4.1, and the outcome indicators included technical success, procedure time, contrast dose, fluoroscopy time, complications, and economic parameters, and subgroup analyses were performed to explore the effects of factors such as patient characteristics and instrument type.

**Results:**

A total of 16 studies were included.There was no significant difference between ICE and TEE in terms of technical success (RR = 1.01,95% CI 1.00–1.02, *P* = 0.24) and total risk of physical complications (RR = 0.94,95% CI 0.82–1.09, *P* = 0.43). Subgroup analysis showed:

**Operative efficiency:**

ICE significantly reduced operative time in the subgroups of single-center studies (MD = −7.28 min, 95% CI: −9.46 to −5.10, *P* < 0.001), with AcuNav catheter (MD = −3.21 min, 95% CI: −6.20 to −0.19, *P* = 0.04), and patients aged <75 years (MD = −15.89 min, 95% CI: −18.95 to −12.82, *P* < 0.001); the use of multi-seal devices was associated with a significant reduction in contrast agent volume (MD = −21.69 mL, 95% CI: −31.44 to −11.94, *P* < 0.001).

**Disease characteristics:**

In the subgroup with a hypertension proportion <90%, ICE shortened both operative time (MD = −12.00 min, 95% CI: −15.08 to −8.92, *P* < 0.001) and fluoroscopic time (MD = −9.32 min, 95% CI: −14.26 to −4.37, *P* = 0.003); however, operative time was prolonged in the ICE group for patients with a proportion of paroxysmal atrial fibrillation ≥50% (MD = 14.20 min, 95% CI: 7.60–20.80, *P* < 0.001).

**Economics:**

ICE reduced professional/anesthesia-related costs (MD = -$2,654, *P* < 0.001) but increased hospitalization costs by approximately 17.8%, with notable geographic heterogeneity in total costs (comparable in the United States, but potentially higher for ICE in China based on existing cost structures). Sensitivity analyses showed good stability of the results, with heterogeneity (I^2^ > 90%) mainly stemming from differences in study design and device type.

**Conclusion:**

The core clinical outcomes (success and safety) of ICE and TEE in LAAO are equivalent, but operational efficiency is moderated by patient age, LV morphology, and device design. ICE is recommended for young, anatomically simple patients at high risk for anesthesia, and individualized decision-making needs to be optimized with team experience and health economic assessment. Future multicenter RCTs and cost-utility analyses are needed to validate long-term benefits.

**Systematic Review Registration:**

PROSPERO CRD42024626272.

## 1 Preamble

Atrial fibrillation (AF), the most common clinical type of cardiac arrhythmia, has a global prevalence of 0.51%. The condition is characterized by irregular contractions of the atria, which predispose to hemodynamic changes leading to thrombosis (1, 2), exposing patients to a significantly higher risk of thromboembolism (3). Although oral anticoagulants are the preferred regimen for the prevention of AF-related stroke and systemic embolism, their clinical use is limited by key issues such as increased risk of bleeding, poor medication adherence, and drug-drug interactions (4, 5). Notably, more than 90% of left heart thrombi in patients with nonvalvular AF originate from the left auricle (2, 6), an anatomical feature that has led to the development of LAAO (LAAO) as an alternative treatment strategy of significant clinical value, particularly in patient populations at high risk of stroke, those who have experienced stroke despite anticoagulant therapy, or those with contraindications to or a high risk associated with long-term oral anticoagulation (4, 7).This interventional technique achieves anatomical isolation of the left auricle through the implantation of a blocking device, thus effectively preventing thrombotic and embolic events (8).

In terms of intraoperative image guidance for LAAO, fluoroscopy combined with transesophageal echocardiography (TEE) is still the gold standard, with the advantages of high-resolution imaging and affordability (9, 10). However, this technique needs to be performed under general anesthesia/deep sedation with tracheal intubation, which not only requires the support of a professional team, but also carries the risk of complications such as aspiration and esophageal injury (6, 11), and may be less tolerated by patients due to invasive operations (5, 9). In recent years, intracardiac ultrasound (ICE) has gained attention as a novel alternative (3, 10, 12), with the greatest advantage that it can be performed under local anesthesia/moderate sedation (5). However, the technique still faces challenges such as limited imaging field of view and high catheter cost. Existing studies have divergent conclusions on the effectiveness of the two guidance modalities in LAAO, and the present study intends to systematically evaluate the differences between ICE and TEE in terms of surgical safety, efficacy, and economy by Meta-analysis method, which will provide an evidence-based basis for clinical decision-making.

## 2 Information and methods

### 2.1 Registration

This study followed the preferred reporting items for systematic reviews and meta-analysis (PRISMA).

PROSPERO 2024 Available from https://www.crd.york.ac.uk/PROSPERO/view/CRD42024626272. Specific registration information can be found in the supplementary file.

### 2.2 Literature search strategy

Search online electronic databases: PubMed, EMbase, Cochrane Library, Web of Science, WanFang Database. Chinese search terms included: intracardiac echocardiography, endocardial echocardiography, cardiac cavity echocardiography, transesophageal echocardiography, left atrial appendage occlusion. English search terms included: intracardiac echocardiography, transesophageal echocardiography, left atrial appendage occlusion, left atrial appendage closure. the search time limit was from the time of construction to February 15, 2025, and references to included studies were retrospectively retrieved for additional access to relevant comparative studies. The specific search process is described in Supplementary File S1.

### 2.3 Inclusion and exclusion criteria

#### 2.3.1 Inclusion criteria

Types of studies: randomized trials (RCTs), cohort studies, and comparative studies such as observational studies.

Study population; patients with AF undergoing LAAO, regardless of age, gender and nationality.

INTERVENTION: ICE-guided LAAO intraoperative imaging was used in the intervention group; TEE-guided LAAO intraoperative imaging was used in the control group.

Outcome metrics: primary outcome metrics included technical success rate, and secondary outcome metrics included procedure time, fluoroscopy time, contrast dose, complication occurrence, and economics-related metrics (including total, professional, hospitalization, and hospitalization costs). The technical success rate refers to successful implantation of the left ear occluder without device-related complications; complications include pericardial effusion/pericardial tamponade, device embolization, peridural leakage of the device, device thrombosis, stroke/TIA, bleeding, and vascular complications; vascular complications include inguinal hematomas, arteriovenous fistulas, pseudoaneurysms, and bleeding at the puncture site (13); and the operative time refers to the time from venous puncture to vascular closure the total time of the procedure. Included studies were required to report at least one primary outcome.

#### 2.3.2 Exclusion criteria

(i) patients with intracardiac thrombus detected by preoperative TEE or CT; (ii) single-arm studies; (iii) repetitive publications; (iv) any type of study where accurate or complete raw data could not be extracted or where data were limited to one patient, such as case reports, case reports, letters, and commentaries; (v) abstracts of conferences, reports of meetings, reviews, laboratory studies in the literature, or experiments on animals; and (vi) studies that were not available due to copyright issues.

### 2.4 Literature screening, data extraction

To ensure the accuracy and completeness of data extraction, we established a standardized data extraction process. According to the established inclusion and exclusion criteria, 2 researchers independently screened the literature, extracted the data and cross-checked them, and any disagreement was resolved through discussion or consultation with a third researcher. Literature was screened by first reading the title and abstract, and then further reading the full text of relevant literature to determine final inclusion. Data extraction mainly extracted: (1) the basic information of the included studies, including the first author, year of publication, country and region, and type of study design; (2) the baseline characteristics of the study population, including the sample size, gender and age of the patients; (3) the characteristics related to the interventions, including the type and location of ICE catheters and the type of left auricular occluder, etc.; (4) the key elements of the evaluation of the risk of bias; and (5) the endpoints and outcomes of concern Measurement data. During the data extraction process, we developed detailed processing principles: for multiple publications of the same study, the most recently published or the version with the most complete data was prioritized as the primary data source. For studies reporting only the median and interquartile spacing or range of outcome effect values, the corresponding formula was used to convert the outcome effect measures to means and standard deviations (14).

### 2.5 Evaluation of the methodological quality of the included studies

In this study, the included literature was independently evaluated by two researchers in strict accordance with the methodological standards of evidence-based medicine, respectively, using internationally recognized risk of bias assessment tools: for observational studies (e.g., cohort studies, case-control studies), ROBINS-I (Risk of Bias Evaluation Tool for Non-Randomized Interventional Studies) scale was used, and seven dimensions, such as confounding factors, selection of study subjects, and classification of interventions were Randomized controlled trials (RCTs) were systematically evaluated according to the risk of bias assessment tool recommended by the Cochrane Handbook version 5.1.0, focusing on 6 core areas, including random sequence generation, allocation concealment, blinding implementation, data completeness and selective reporting. A double-blind cross-validation mechanism was implemented in the evaluation process, and after the researchers completed the initial evaluation independently, disagreements were resolved through structured discussions, and a third methodology expert was introduced for arbitration when necessary.

### 2.6 Statistical analysis

Meta-analysis of extracted data was analyzed using RevMan 5.4.1 provided by the Cochrane Collaboration.Meta-analyses and individual study estimates were presented in forest plots. Mean difference (MD) and 95% confidence interval (CI) were used for measurement data, and relative risk (RR) and 95% CI were used for count data. All outcome data were processed using a random effects model.Heterogeneity between studies was assessed using chi-square and I^2^ tests. Studies were considered homogeneous if *P* > 0.1 and I^2^ ≤ 50%; conversely, studies were heterogeneous if *P* ≤ 0.1 and I^2^ > 50%, and sensitivity analyses and subgroup analyses were performed on the sources of heterogeneity and factors affecting the posterior values of the combined effects. Subgroup analyses were predefined based on clinical relevance and data distribution: age was dichotomized at 75 years, a common geriatric threshold; hypertension prevalence at 90%, approximating the median in included studies; and paroxysmal AF proportion at 50% to distinguish predominantly paroxysmal from non-paroxysmal populations. Sensitivity tests were used to determine whether analytic heterogeneity would affect the stability of the results by comparing the effect sizes of the random-effects model and the fixed-effects model. To identify potential publication bias and small-sample effects, we used a multidimensional assessment approach: first, the symmetry of the funnel plot was assessed by the visual method, focusing on the distribution of small-sample studies at the bottom of the graph, with the significance level set at α = 0.05. In addition to visual inspection of funnel plots, statistical assessment for potential publication bias was performed using Egger's regression test for outcomes with ≥10 studies, as this test is underpowered with fewer studies. For outcomes with fewer than 10 studies, publication bias was evaluated qualitatively based on funnel plot symmetry.

### 2.7 Grading the quality of evidence (GRADE)

The overall quality of evidence for each primary and key secondary outcome was assessed using the Grading of Recommendations Assessment, Development, and Evaluation (GRADE) framework. The initial quality rating for randomized trials is high and for observational studies is low. The quality was then downgraded based on predefined criteria: risk of bias (study limitations), inconsistency (heterogeneity), indirectness, imprecision (wide confidence intervals), and publication bias. The evidence quality was categorized as high, moderate, low, or very low.

## 3 In the end

### 3.1 Literature search results

**Figure 1:**
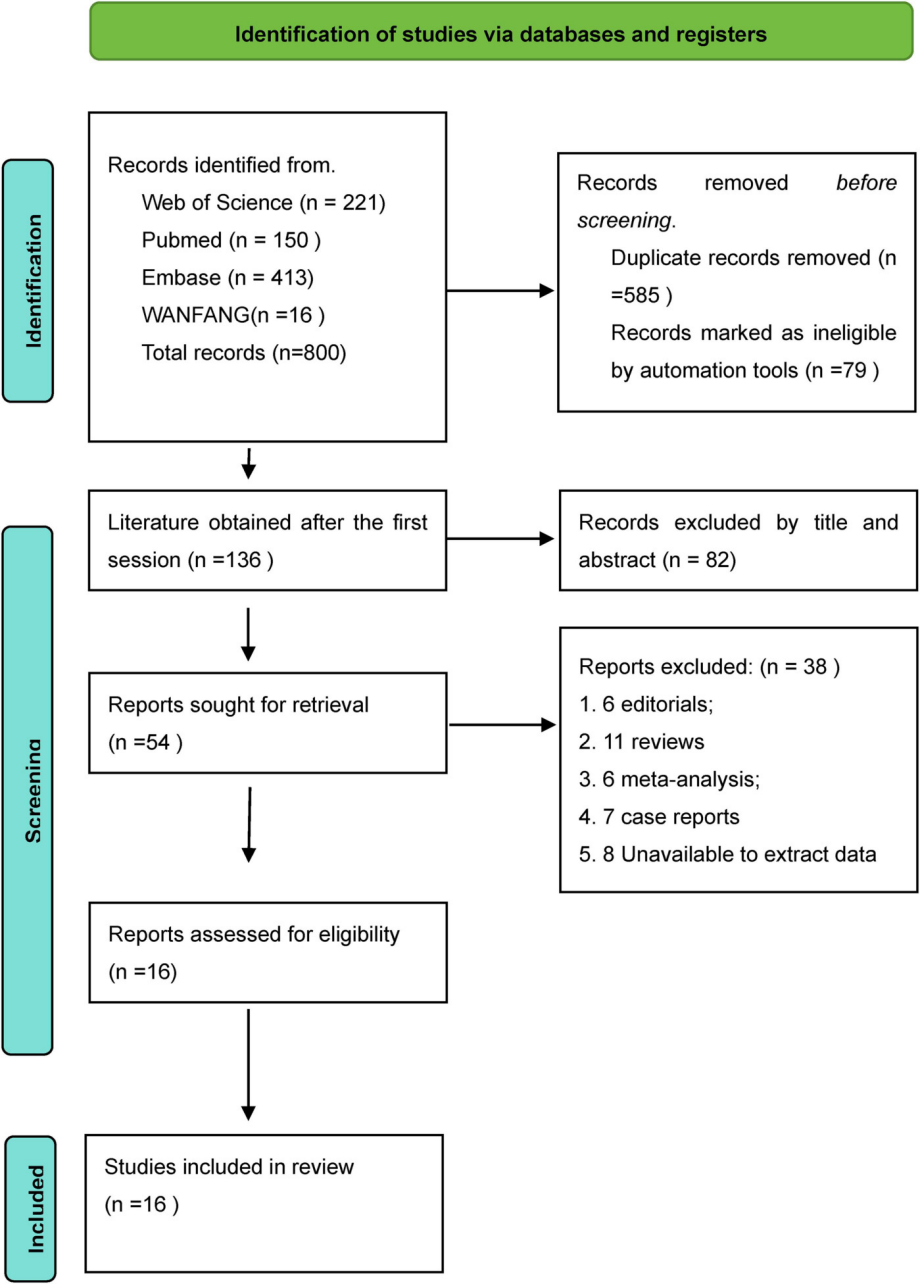
Revman search flowchart.

A total of item studies were retrieved in our initial search, of which 150 were PubMed, 221 Web of Science, 10 Cochrane Library, 413 Embase, and 16 WANFANG, and we identified 136 records after deleting duplicates and 664 academic papers. Subject irrelevance was excluded from 82 based on screening of titles and abstract reviews, and the full text of 54 articles was subsequently reviewed. The final result was 16 (3, 6, 10, 15–27) studies that met the inclusion criteria were eligible for data extraction and quantitative analysis. The specific process is shown in Figure 1.

### 3.2 Results of included study characteristics

A total of 16 clinical studies on LAAO (ICE/TEE) were included in this study, and their basic characteristics are summarized as follows (see Table 1): predominantly single-center studies (10), including 8 prospective cohort studies and 8 retrospective cohort studies; and 6 multicenter studies. Sample sizes varied significantly, ranging from 23 cases (Streb 2019) to 2,603 cases (Su 2022), with an uneven distribution of sample sizes between the ICE and TEE groups (e.g., in Su 2022, there were only 95 cases in the ICE group, while the TEE group reached 2,508 cases). The percentage of males overall ranged from 45.5% (Streb 2019 ICE group) to 81% (Frangieh 2017 ICE group), with the majority of studies ranging from 55% to 70% male. The mean age of the subjects was clustered between 70 and 75 years (standard deviation ± 7–9 years), with significant age differences in some studies (e.g., up to 80.3 years in the TEE group in Frangieh 2017). Follow-up time spanned a wide range, from a minimum of 1 month (Streb 2019, Su 2022) to a maximum of 12 months (Nielsen-Kudsk 2019, Kim 2018, et al.), and some studies did not report on the duration of follow-up (NA). Device types such as blockers: predominantly Watchman (9), Amulet/ACP (8), some studies used LAmbre (e.g., Du Xianfeng 2021). ICE catheters: AcuNav (7) and ViewFlex Xtra (6) were the commonly used models, but catheter type was not specified in 5 studies (NA). Paroxysmal AF: wide range of prevalence (17.42%–81.6%), not reported in some studies (NA). Hypertension: most studies accounted for more than 80% (e.g., 92.2% in Alkhouli 2020), but only about 57% in Morcos 2022.

**Table 1:** List of basic characteristics of the included literature.

Included studies/publication dates	Research design	Sample size (ICE/TEE, cases)	Percentage of males (ICE/TEE, %)	Age (ICE/TEE, years)	Follow-up time (months)	Types of Left Ear Occluders	ICE Catheter Types	Percentage of paroxysmal AF (ICE/TEE, %)	Percentage of hypertension (ICE/TEE, %)
Kim 2018 (3)	Multiple, retrospective	144 (41/103)	58.5/49.5	71.4 ± 9.3/72.3 ± 9.2	1.5, 6, 12	Amulet, ACP, Watchman	AcuNav	34.1/27.3	90.2/83.5
Alkhouli 2020 (6)	Single, retrospective	286 (90/196)	62.8/55.6	75.7 ± 8.01/75.2 ± 7.8	NA	Watchman	AcuNav, ViewFlex Xtra	NA	92.2/87.2
Berti 2018 (10)	Multiple, retrospective	604 (187/417)	66/65	76 ± 8/74 ± 7	6–12	Amulet, ACP	AcuNav	32/36	NA
Frangieh 2017 (15)	Single, forward-looking	76 (32/44)	81/57	74.6 ± 9.3/80.3 ± 7.7	NA	Watchman	AcuNav	69/54	84/86
Korsholm 2017 (16)	Single, retrospective	216 (109/107)	73.8/62.4	73 ± 7.8/73 ± 9.7	2	Amulet, ACP	ViewFlex Xtra	54.1/49.5	83/80
Reis2018 (17)	Single, retrospective	82 (26/56)	64.6	74 ± 8	3, 6, 9, 12	Amulet, ACP, Watchman	NA	35.4	NA
Nielsen-Kudsk 2019 (18)	Many, forward-looking	1,085 (130/955)	60/65	75 ± 8/75 ± 9	12	Amulet	ViewFlex Xtra	NA	NA
Streb 2019 (19)	Single, forward-looking	23 (11/12)	45.5/33.3	77/73^a^	1	Amulet	AcuNav	45.5/66.6	81.82/91.67
Hemam 2019 (20)	Multiple, retrospective	104 (53/51)	62.3/60.8	77 ± 10/76 ± 7	1.5, 4	Watchman	SoundStar, ViewFlex Xtra	NA	81/90
Du Xianfeng 2021 (21)	Single, forward-looking	172 (36/136)	63.9/65.4	70.3 ± 8.7/68.4 ± 7.6	1.5, 3, 6, 12	ACP, Watchman, LAmbre	SoundStar	27.8/30.1	NA
Gianni 2021 (22)	Single, forward-looking	190 (122/68)	66/60	72 ± 8/75 ± 9	2 (1.5–4)	Watchman	NA	NA	NA
Pommier 2021 (23)	Single, forward-looking	224 (175/49)	69.7/71.4	76 ± 8/75 ± 7	1.5–2	ACP, Watchman	ViewFlex Xtra	30/28	91/96
Morcos 2022 (24)	Multiple, retrospective	790 (395/395)	59.5/68.3	70.7 ± 0.64/70.4 ± 0.65	NA	NA	NA	NA	59.4/57.1
Su 2022 (25)	Many, forward-looking	2,603 (95/2 508)	57.6	69.1 ± 9.4	1	Watchman	NA	81.6	68.8
Qian 2024 (26)	Single, forward-looking	132/132	57.58/60.61	62.98 ± 8.14/62.44 ± 8.52	NA	Watchman, LAmbre	AcuNav	27.27/17.42	62.88/66.67
André Grazina 2023 (27)	Single, forward-looking	45/43	71.1/65.1	75.5 ± 9.6/74.2 ± 9.8	NA	ACP or Amulet, WATCHMAN and LAmbre	NA	NA	68.9/83.7

^a^ Denotes median; single: single center; multiple: multicenter; prospective: prospective cohort study; retrospective: retrospective cohort study; NA, not applicable.

### 3.3 Literature quality evaluation results

The results of ROBINS-I evaluation showed the following: confounding bias: 10 low-risk, 5 medium-risk, and 2 high-risk; selection bias of the study population: 15 low-risk, and only 1 in both medium-risk (Nielsen-Kudsk 2019) and high-risk (Du Xianfeng 2021); bias of intervention categorization: all studies were “low” risk, indicating that the interventions were clearly defined and accurately categorized; bias of deviation from established interventions: all studies (100%) were at low risk of this bias, and all included studies strictly followed the established protocols in implementing the interventions without significant deviation; bias of missing data: the vast majority of the studies performed well in terms of data completeness, with only 1 study Bias of missing data: the vast majority of studies performed well in terms of data completeness, with only 1 study rated as medium risk for potential problems; bias of outcome measures: all studies were “low” risk, suggesting that outcome measures were consistent and reliable; bias of selective reporting of outcomes: 12 were low risk, 4 were medium risk, and 2 were high risk. The majority of studies (9) had a “medium” overall risk of bias and needed to interpret their results with caution; 4 studies were of high quality (overall bias “low”) and had high confidence in their results; 3 studies (Reis 2018, Su 2022) had a “high” overall risk of bias and had a “high” overall risk of bias, indicating consistent and reliable outcome measures. “high” and their findings need to be treated with a high degree of caution. See Table 2.

**Table 2:** ROBINS-I quality evaluation table.

Nathaniel's study	confounding bias	Subject selection bias	Bias in classification of interventions	Bias from established interventions	Missing data bias	Bias in outcome measurements	Bias in selective reporting of results	overall bias
Kim 2018 (3)	Low	Low	Low	Low	Low	Low	Middle	Medium
Alkhouli 2020 (6)	Medium	Low	Low	Low	Low	Low	Low	Medium
Berti 2018 (10)	Low	Low	Low	Low	Low	Low	Low	Low
Frangieh 2017 (15)	Medium	Low	Low	Low	Low	Low	Low	Medium
Korsholm 2017 (16)	Medium	Low	Low	Low	Low	Low	Medium	Medium
Reis2018 (17)	High	Low	Low	Low	Low	Low	High	High
Nielsen-Kudsk 2019 (18)	Medium	Medium	Low	Low	Medium	Low	Low	Medium
Streb 2019 (19)	Medium	Low	Low	Low	Low	Low	Low	Medium
Hemam 2019 (20)	Low	Low	Low	Low	Low	Low	Low	Low
Du Xianfeng 2021 (21)	Medium	High	Low	Low	Low	Low	Low	High
Gianni 2021 (22)	Medium	Low	Low	Low	Low	Low	Low	Medium
Pommier 2021 (23)	Medium	Low	Low	Low	Low	Low	Medium	Medium
Morcos 2022 (24)	Low	Low	Low	Low	Low	Low	Low	Low
Su 2022 (25)	High	Low	Low	Low	Low	Low	Low	High
Qian 2024 (26)	Low	Low	Low	Low	Low	Low	Medium	Medium
André Grazina 2023 (27)	Low	Low	Low	Low	Low	Low	Low	Low

Low, low risk; Medium, moderate risk; High, high risk.

### 3.4 Meta-analysis results

We extracted the data relevant to this paper for comparison by reading these 16 papers for Meta-analysis with the primary outcome as technical success, secondary outcomes as procedure time, contrast dose, device fluoroscopic time, and complication occurrence as outcome metrics, and subgroup analysis was used to reveal potential determinants of LAAO outcomes between the ICE and TEE groups, respectively. A total of eight subgroup factors were identified based on the characteristics of the eligible studies, including study design, age cutoffs, sample size in the ICE group, type of AF, proportion of males, proportion of hypertension, type of device, and type of catheter use. A multicenter subgroup was defined if the study design included more than one center; otherwise, a single-center subgroup was defined. Two subgroups, including ≥75 years and <75 years, were categorized according to an age cutoff value of 75 years. Patients were categorized as ≥50% PAF if more than 50% had paroxysmal AF (PAF); otherwise, they were categorized as <50% PAF. based on the proportion of males, they were categorized as ≥70% and <70%. Similarly, hypertensive proportions ≥90% and <90% were defined separately. Based on sealing position, available sealers can be broadly categorized into plug and disk type. Plug sealers, also known as single sealers, include Watchman, Plato, and Lefort. disk sealers, also known as dual sealers, include ACP, Lambre, Lacbes, and Leftear. If the LAAO device includes only dual sealers, they are categorized under the dual sealers subgroup. If the LAAO device includes only single sealers, they are categorized under the single sealers device grouping. Depending on the type of ICE catheter, the group is divided into AcuNav®, ViewFlex Xtra®, SoundStar®, and a combined group where different types of catheters are used in combination. See supplementary file 2.

#### 3.4.1 Main outcome indicators

##### 3.4.1.1 Technical success rate

**Figure 2:**
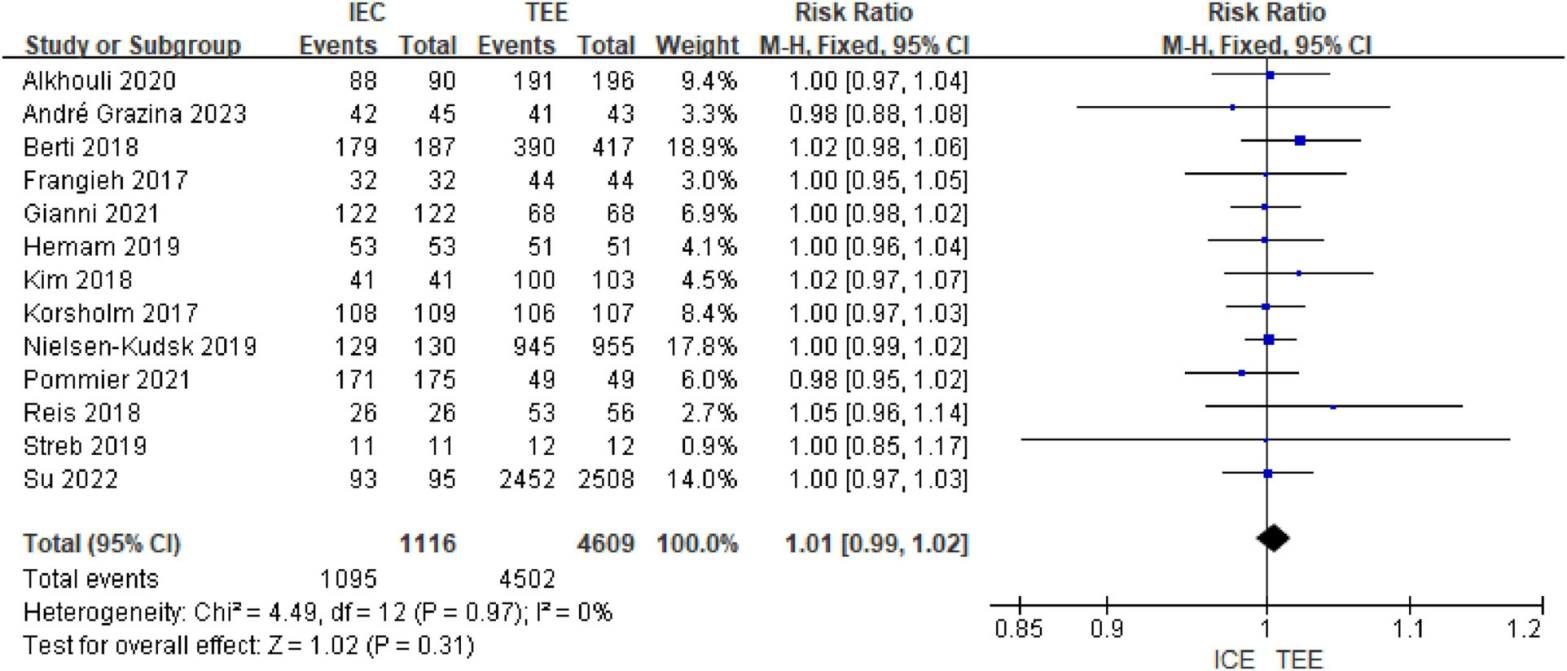
Forest map of technical success rates.

A total of 13 studies were included, containing 1116 patients in the ICE group and 4,609 patients in the TEE group. Four of the studies showed a 100% technical success rate in both the ICE and TEE groups. Significant heterogeneity was observed (I2 = 0%, *P* = 0.97), and the results of the fixed-effects model Meta-analysis of all the included studies showed no statistically significant difference between the ICE and TEE groups [RR = 1.01, 95% CI (1.00, 1.02), *P* = 0.24]; See Figure 2.

In all subgroup analyses, there was no significant difference in the final success rate of ICE vs. TEE (all RR values were close to 1, and 95% confidence intervals included 1), and the *P* value for the interaction between subgroups was >0.05, indicating that different subgrouping factors (e.g., study design, sample size, patient characteristics, etc.) did not significantly affect the results. The index of heterogeneity (I^2^) for all studies was 0%, indicating a high degree of consistency in the results across studies and high reliability of conclusions. Subgroup-specific analyses:Study design: there was no difference in the success rate between single-center and multicenter studies (*P* = 0.37); sample size: sample size of ≤100 vs. >100 did not affect the results (*P* = 0.87); patient characteristics: proportion of males (*P* = 0.25), age demarcation (*P* = 0.80), proportion of hypertension (*P* = 0.74), and proportion of paroxysmal AF (*P* = 0.63) did not significantly affect success rates; device type: success rates were similar for different blocking devices (dual-seal, single-seal, multi-seal) or catheter types (AcuNav, ViewFlex, integrated) (*P* = 0.75 and *P* = 0.44). The available evidence suggests that there is no statistically significant difference between the success rates of ICE and TEE in LAAO and that both can be used as effective guidance modalities. See supplementary file 3.

#### 3.4.2 Secondary outcome indicators

##### 3.4.2.1 Duration of surgery

**Figure 3:**
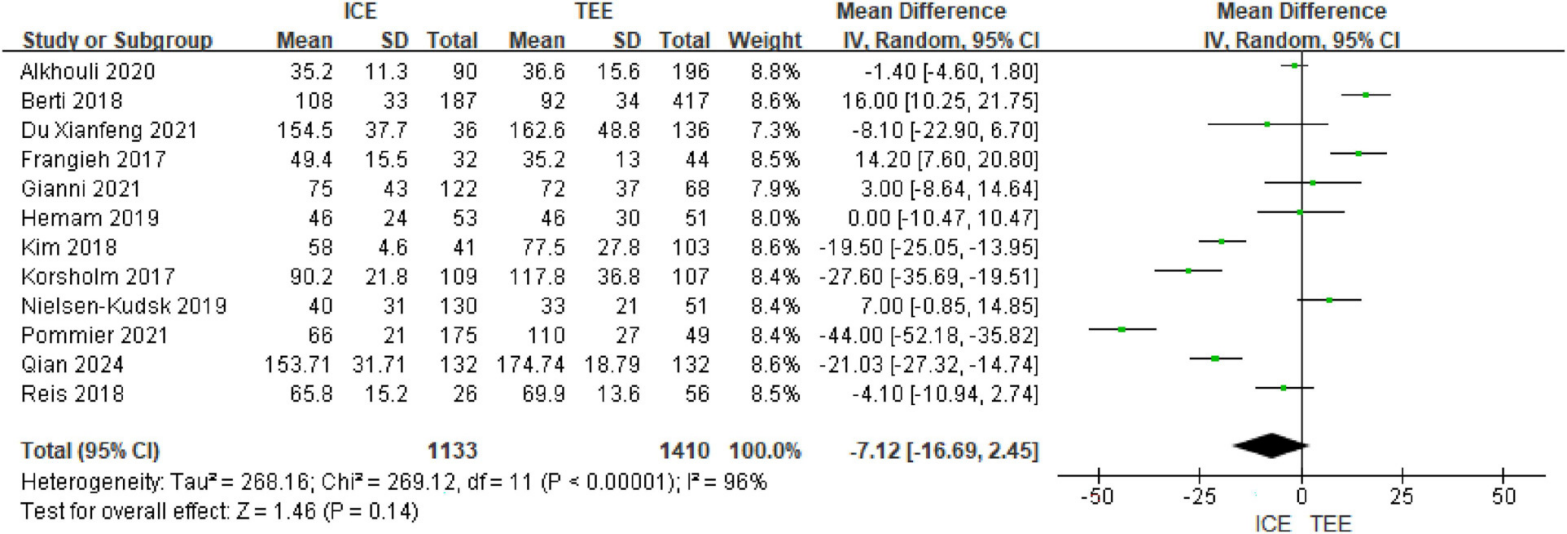
Surgical time forest map.

A total of 12 clinical studies provided total operative time and significant heterogeneity was observed (I2 = 96%, *P* < 0.00001). Meta-analysis of the included studies using a random-effects model showed similar data on total operative time between groups (MD = −7.12; 95% CI: −16.69, 2.46; *P* = 0.14) See Figure 3.

Subgroup analyses revealed several important moderating effects. ICE significantly shortened operative time in the subgroups of single-center studies (MD = −7.28 min, 95% CI: −9.46 to −5.10, *P* < 0.001), multi-seal device use (MD = −19.75 min, 95% CI: −22.93 to −16.56, *P* < 0.001), and patients aged <75 years (MD = −15.89 min, 95% CI: −18.95 to −12.82, *P* < 0.001). Conversely, operative time was prolonged with ICE in the subgroup with a proportion of paroxysmal AF ≥50% (MD = 14.20 min, 95% CI: 7.60–20.80, *P* < 0.001). Detailed subgroup results are available in supplementary file 3.

Subgroup analyses showed significant differences between ICE and TEE in operative time for LV occlusion, and the direction of the differences was closely related to subgroup characteristics. Significant *P* values for the interaction of multiple subgroups (e.g., study design, sample size, and proportion of AF) indicated that subgroup factors had a significant effect on the results. Subgroup-specific analyses.

Study design: single-center vs. multicenter: ICE significantly shortened operative time in single-center studies (MD = −7.28,*P* < 0.00001), while the difference was not significant in multicenter studies (MD = −0.40,*P* = 0.82). Sample size: ICE reduced operative time by a greater magnitude with a sample size >100 (MD = −9.07 vs.−3.02). Patient characteristics: proportion of men <70%: ICE shortened time (MD = −3.42, *P* = 0.0001), whereas the difference was not significant at ≥70% (MD = 0.67, *P* < 0.00001); age <75 years: ICE significantly shortened time (MD = −15.89, *P* < 0.00001), and the difference at ≥75 years was not statistically significant (MD = −12.11, *P* = 0.56); proportion of AF ≤50%: ICE shortened time (MD = −13.07, *P* < 0.00001), whereas TEE time was shorter in >50% (MD = 14.20, *P* < 0.00001). Device and catheter type: multiseal mechanism devices: significantly shorter time to ICE (MD = −19.75, *P* < 0.00001); ViewFlex catheters: shorter time to TEE (MD = 20.80,*P* < 0.00001) and the opposite for AcuNav catheters (MD = −3.21, *P* = 0.04). Heterogeneity was extremely high in most subgroups (I^2^ > 90%), suggesting large inter-study differences, but the differences between subgroups were statistically significant in all subgroups (*P* < 0.05), suggesting that the groups were responsible for the heterogeneity of the results. See supplementary file 4.

##### 3.4.2.2 Contrast agent dose

**Figure 4:**
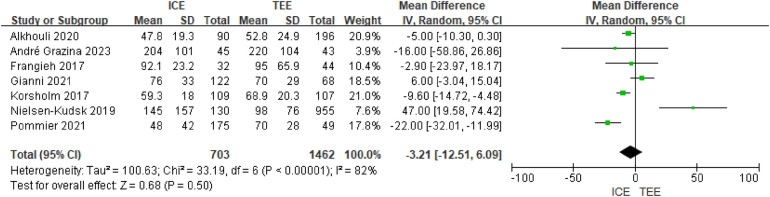
Forest map of contrast agent doses.

A total of 7 eligible studies reported contrast agent doses. Significant heterogeneity was observed (I2 = 82%, *P* < 0.00001). Meta results of the included studies using a random-effects model showed no significant difference in the use of contrast agent dose in the ICE group compared with the TEE group (MD = −3.21; 95% CI: −12.51, 6.09; *P* = 0.50). See Figure 4.

Subgroup analyses indicated that ICE guidance significantly reduced contrast dose under specific conditions. This advantage was primarily observed in subgroups using multi-seal devices (MD = −21.69 mL, 95% CI: −31.44 to −11.94, *P* < 0.001), patients aged <75 years (MD = −9.69 mL, 95% CI: −14.77 to −4.61, *P* = 0.0002), and with a male proportion ≥70% (MD = −11.76 mL, 95% CI: −16.21 to −7.30, *P* < 0.001). Detailed subgroup results are available in supplementary file 4.

Subgroup analyses showed significant differences between ICE and TEE in contrast dose for LAAO, but the direction and magnitude of the differences varied by subgroup characteristics. *P* values for interactions were significant for multiple subgroups (e.g., study design, proportion of men, device type), indicating that subgrouping factors had a significant effect on outcomes. Subgroup-specific analyses. See supplementary file 5.

Study design: significant dose reduction by ICE in a single-center study (MD = −7.15, *P* < 0.00001); multicenter study showed an increase in dose by ICE (MD = 47.00, *P* = 0.0008), with a difference between the groups of *P* = 0.0001; Sample size: dose reduction by ICE was more pronounced with a sample size of >100 (MD = −7.29 vs. −5.03), but the difference between groups was not significant (*P* = 0.50). Patient characteristics: proportion of males ≥70%: significant dose reduction by ICE (MD = −11.76, *P* < 0.00001) compared to no difference at <70% (*P* = 0.65), with a between-group difference of *P* = 0.0009; age cut-offs: significant dose reduction at both <75 and ≥75 years (MD = −9.69 and −6.95), but not significant (*P* = 0.43) between-group difference significant (*P* = 0.43); proportion of AF ≤50%: significant dose reduction for ICE (MD = −12.17, *P* < 0.00001), whereas no difference was observed for >50% (*P* = 0.79). Devices and catheter types: multiseal mechanism devices: significant dose reduction by ICE (MD = −21.69, *P* < 0.0001), with a difference between groups of *P* = 0.001; ViewFlex catheters: significant dose reduction by ICE (MD = −10.58, *P* < 0.00001), but nonsignificant results for the AcuNav and the integrated catheters and but a difference between groups of differences were not significant (*P* = 0.26).

##### 3.4.2.3 Fluoroscopic time

**Figure 5:**
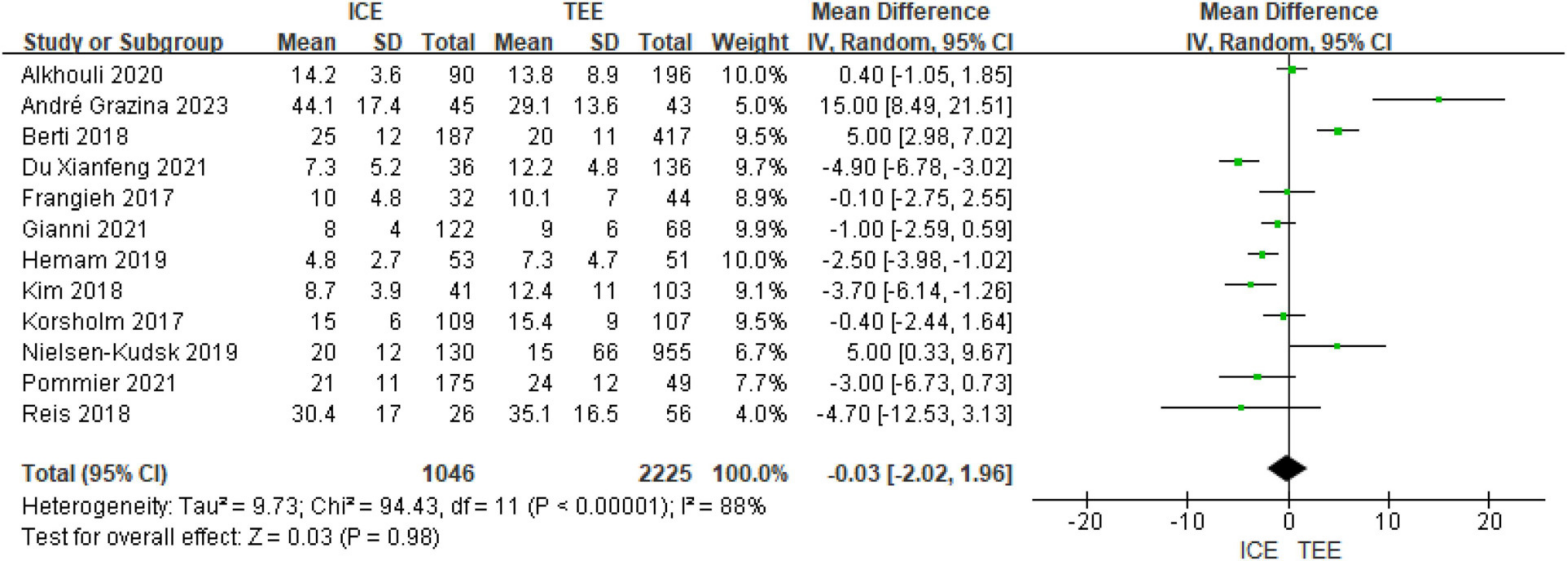
Forest map of fluoroscopy times.

A total of 12 eligible studies reported fluoroscopy times, and significant heterogeneity was observed (I2 = 88%, *P* < 0.00001). The included studies used a random-effects model,and the pooled results showed that fluoroscopy time in the ICE group was significantly comparable to that in the TEE group (MD = −0.03; 95% CI: −2.02, 1.96; *P* = 0.98).See Figure 5.

Subgroup analyses showed that the advantage of ICE in reducing fluoroscopy time was closely associated with patient and device characteristics. Fluoroscopy time was significantly shorter with ICE in subgroups of patients aged <75 years (MD = −1.98 min, 95% CI: −2.92 to −1.03, *P* < 0.001), using multi-seal devices (MD = −3.46 min, 95% CI: −4.79 to −2.13, *P* < 0.001), and with the SoundStar catheter (MD = −4.90 min, 95% CI: −6.78 to −3.02, *P* < 0.001). Detailed subgroup results are available in supplementary file 5.

Subgroup analyses showed a significant difference between ICE and TEE in fluoroscopic time for left ear occlusion, but the direction and magnitude of the difference varied by subgroup characteristics. Significant *P* values for the interaction of multiple subgroups (e.g., sample size, age demarcation, device type, catheter type) indicated that subgrouping factors had a significant effect on the results. Subgroup-specific analyses. See supplementary file 5.

Study design: in the single-center study, ICE significantly shortened fluoroscopic time (MD = −1.00, *P* = 0.01), whereas the difference in the multicenter study was not significant (MD = 0.33, *P* = 0.53), and the difference between the groups was *P* = 0.32. Sample size: with a sample size of ≤100, ICE significantly shortened duration (MD = −1.72, *P* < 0.0001), whereas no significant difference at 100 (MD = −0.75, *P* = 0.14), and the difference between groups was *P* = 0.0002.

Patient characteristics: proportion of males ≥70%: ICE significantly shortened duration (MD = −1.61, *P* = 0.003), whereas there was no difference at <70% (*P* = 0.47), and the difference between the groups was *P* = 0.05. Age <75 years: ICE significantly shortened duration (MD = −1.98, *P* < 0.0001), and the difference was not significant at ≥75 years (MD = −0.19, *P* = 0.65), and the difference between groups was *P* = 0.0007; proportion of hypertension <90%: ICE significantly shortened the duration (MD = −9.32, *P* = 0.003), and there was no difference at ≥90% (*P* = 0.95), and the difference between groups was not significant (*P* = 0.0.09); proportion of AF ≤50%: ICE significantly reduced the dose (MD = - 1.14,*P* = 0.02), whereas there was no difference at >50% (*P* = 0.94) and the difference between groups was not significant (*P* = 0.47). Device and catheter type: multi-seal mechanism devices: ICE significantly shortened duration (MD = −3.46, *P* < 0.00001), whereas single-seal mechanism had superior TEE (MD = 2.56, *P* = 0.0002), with a significant between-group difference between devices (*P* < 0.0001); SoundStar catheters: ICE significantly shortened duration (MD = −4.90, *P* < 0.00001), other catheter types had insignificant or nearly significant differences, with significant differences between groups (*P* < 0.00001).

##### 3.4.2.4 Summarize security results

**Figure 6:**
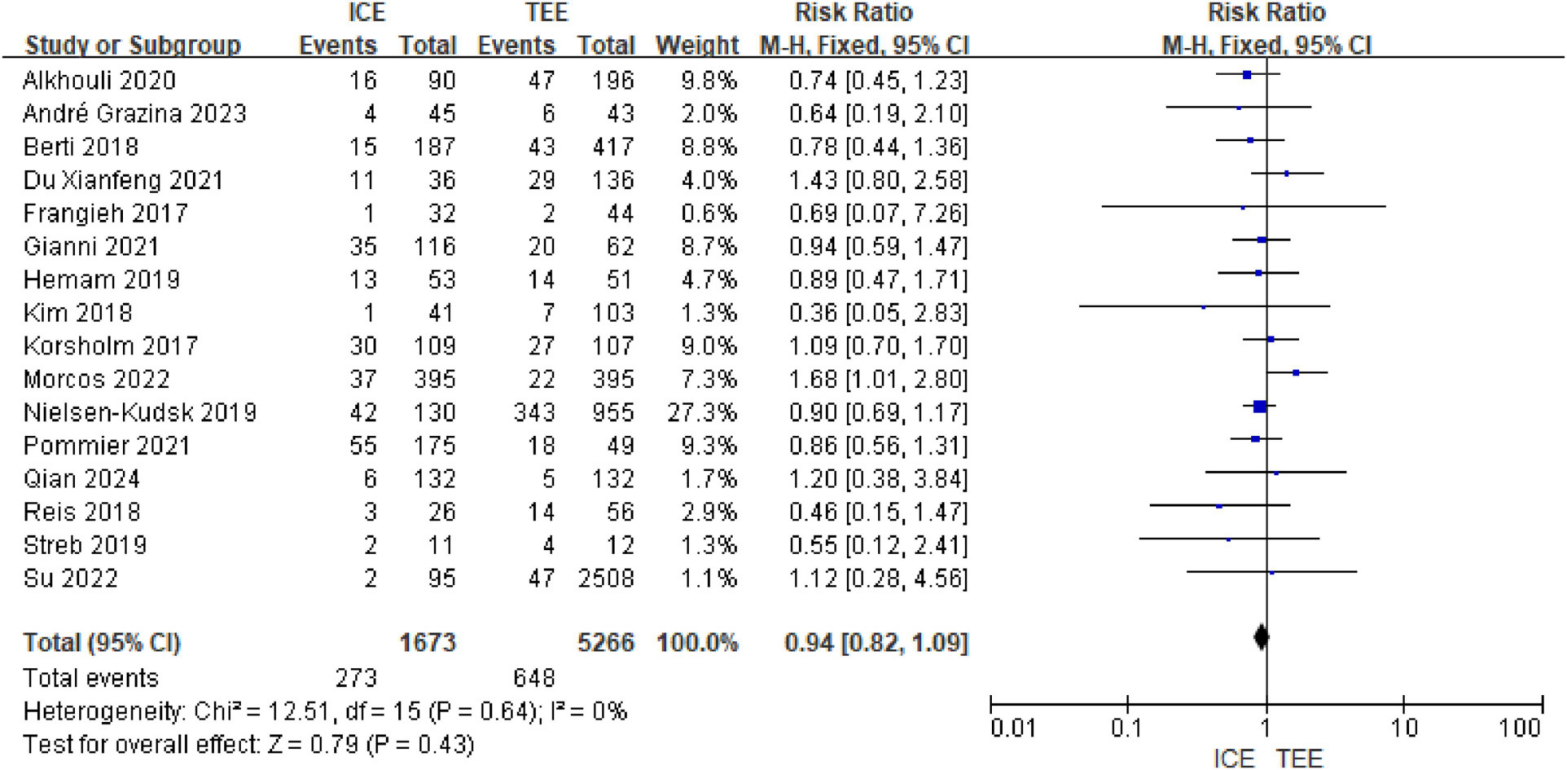
Forest map of complications.

A total of 16 studies were included, containing 1,673 patients in the ICE group and 5,266 patients in the TEE group. Meta-analysis of the fixed-effects model of the included studies showed that the difference in complication rates between the ICE group and the TEE group was not statistically significant [RR = 0.94, 95% CI (0.82, 1.09), *P* = 0.43]. See Figure 6.

The available evidence suggests that there is no significant difference in the risk of complications between ICE and TEE for LAAO, and that both have comparable safety profiles, and a summary categorization of available complications yielded pericardial effusion/pericardial tamponade, device embolism, peripheral leakage of the device, device thrombosis, stroke/TIA, hemorrhage, and vascular complications.There was no statistically significant difference between ICE and TEE in the rate of complications of LAAO (95% confidence intervals for all RR values included 1 and all *P* values > 0.05). None of the *P* values for the interactions for each complication were significant (all ≥0.18), suggesting consistent effects across subgroups. See Table 3.

**Table 3:** Table of specific complication occurrences.

Types of complications	Numbers of study	RR (95% CI)	I^2^(%)	*P* value	*P* for interaction
Pericardial effusion/pericardial tamponade	12	1.48 (0.96,2.26)	0	0.07	0.42
device embolism	10	0.87 (0.31,2.41)	0	0.79	
Leakage around the unit	8	0.99 (0.78,1.26)	0	0.95	
device thrombosis	7	0.76 (0.32,1.80)	0	0.53	
Stroke/TIA	12	1.10 (0.67,1.81)	0	0.70	
hemorrhage	10	0.77 (0.52,1.13)	0	0.18	
Vascular complications	7	1.28 (0.61,2.69)	0	0.51	

##### 3.4.2.5 Economics-related indicators

A total of 3 included studies reported on metrics related to the economics of ICE and TEE, including total costs, professional costs (e.g., ultrasound catheterization fees specific to ICE and anesthesia costs specific to TEE, etc.), hospitalization costs, and inpatient costs. Among these, regarding professional costs, both 2 studies showed that the ICE group was significantly lower than the TEE group. For inpatient costs and hospitalization costs, Morcos et al. and Alkhouli et al. found the ICE group to be significantly higher than the TEE group, whereas Hemam et al. found both groups to be similar. For total costs, Alkhouli et al. found no significant difference between the two groups, while Hemam et al. found the TEE group to be significantly higher; see Table 4.

**Table 4:** Table of results for economics-related indicators.

nnEconomics-related indicators	Inclusion of studies	Sample size analyzed (ICE/TEE, examples)	*P*-value	Average cost (United States dollars)	
				ICE	TEE
Hospitalization costs	Morcos 2022	790 (395/395)	<0.001	34826	29,563
	Hemam 2019	20 (10/10)	0.117	32,290	31,482
Hospital fees	Alkhouli 2020	286 (90/196)	<0.001	76,366	71,114
	Hemam 2019	20 (10/10)	0.396	128,275	129,733
Professional fees	Alkhouli 2020	286 (90/196)	<0.001	2,654	6,033
	Hemam 2019	20 (10/10)	<0.001	4,267	11736
Total cost	Alkhouli 2020	286 (90/196)	0.15	79,020	77147
	Hemam 2019	20 (10/10)	<0.001	132,202	141,468

### 3.5 Sensitivity analysis

To assess the stability of the results of Meta-analysis, this study conducted a sensitivity test by eliminating sources of heterogeneity. To address the heterogeneous differences in outcome indicators in the included studies, the data were analyzed using fixed-effects model and random-effects model, respectively, and the robustness of the conclusions was verified by comparing the combined effect sizes and confidence interval ranges of the risk factors under the two models. The results showed that:

Consistency of effect sizes: The combined effect sizes of the risk factors in the fixed-effects and random-effects models were close to each other, and the effect sizes obtained in the fixed-effects model were all within the confidence interval of the random-effects model, indicating that there were no significant differences between the different models.

STABILITY ASSESSMENT: The above results suggest that the Meta-analysis conclusions are less sensitive to model selection, the data stability is good, and the effect of heterogeneity on the overall conclusions is controllable. In summary, the sensitivity analysis further supports the robustness of this study, and no conclusion bias due to model switching was found, suggesting that the current combined results have high reliability. see Table 5.

**Table 5:** Sensitivity analysis.

Outcome indicator	Effect model	Effect size	95% confidence interval	Effect model	Effect size	95% confidence interval
Technical success rate	FE	RR = 1.01	(0.99,1.02)	RE	RR = 1.00	(0.99,1.01)
Surgical time	FE	MD = −5.24	(−7.08,−3.41)	RE	MD = −7.12	(−16.69,2.45)
Contrast medium dose	FE	MD = −6.43	(−9.59,−3.27)	RE	MD = −3.21	(−12.51,6.09)
Fluoroscopy times	FE	MD = −0.76	(−1.38,−0.13)	RE	MD = −0.03	(−2.02,1.96)
Complications	FE	RR = 0.94	(0.82,1.09)	RE	RR = 0.95	(0.82,1.09)

FE, fixed effects model; RE, random effects model.

### 3.6 Publication bias

**Figure 7:**
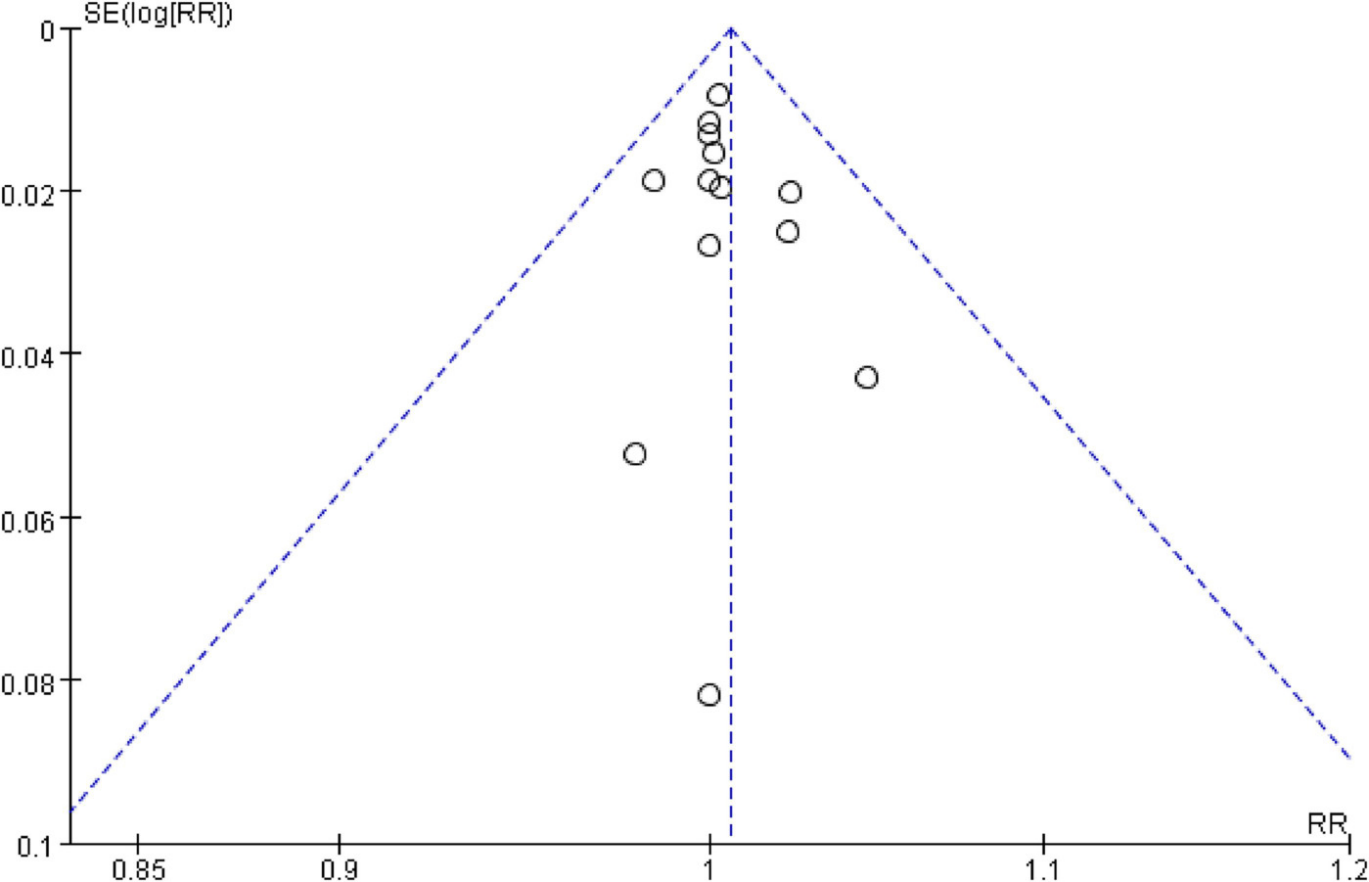
Technology success rate funnel chart.

**Figure 8:**
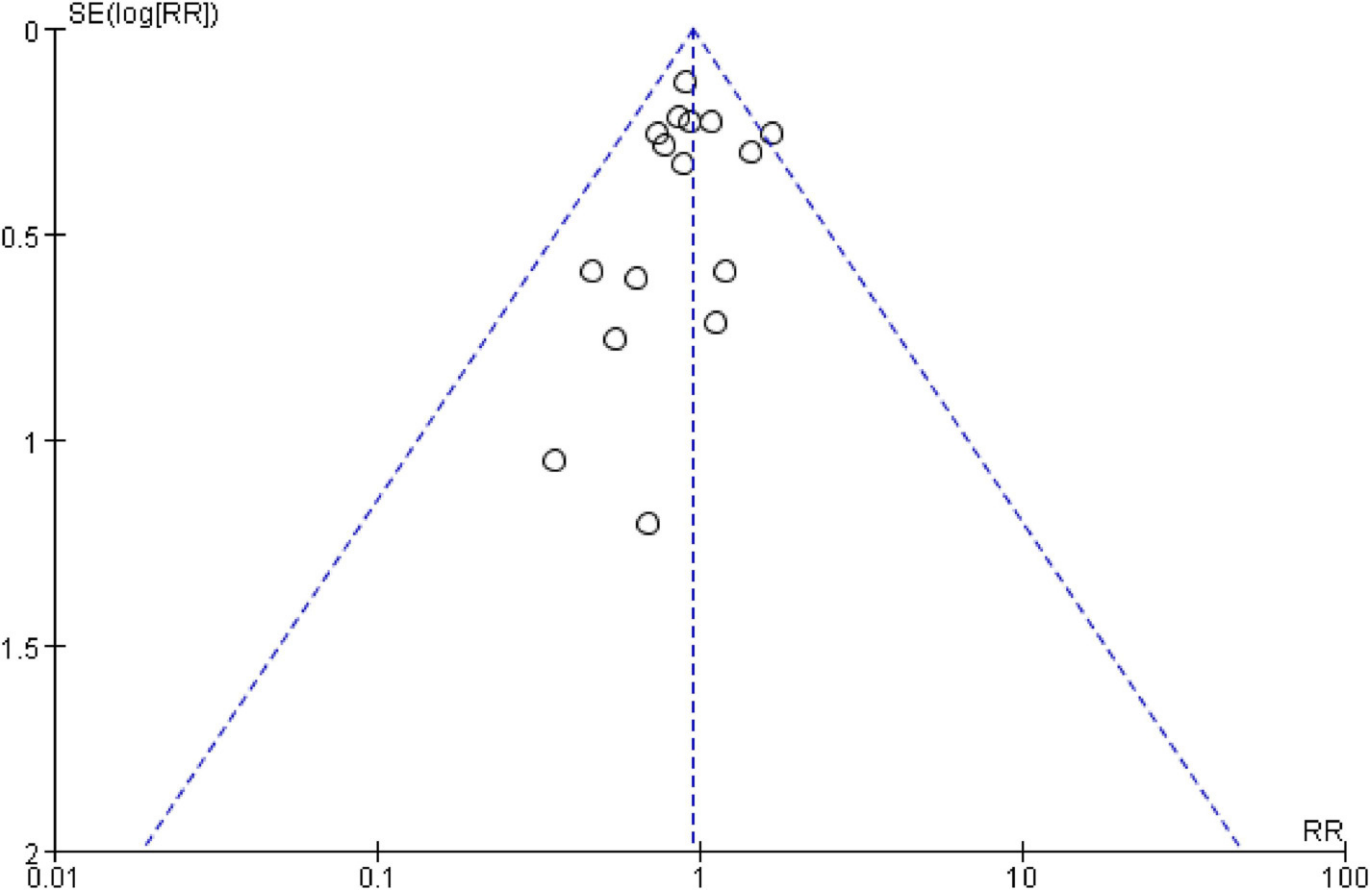
Funnel diagram of complications.

**Figure 9:**
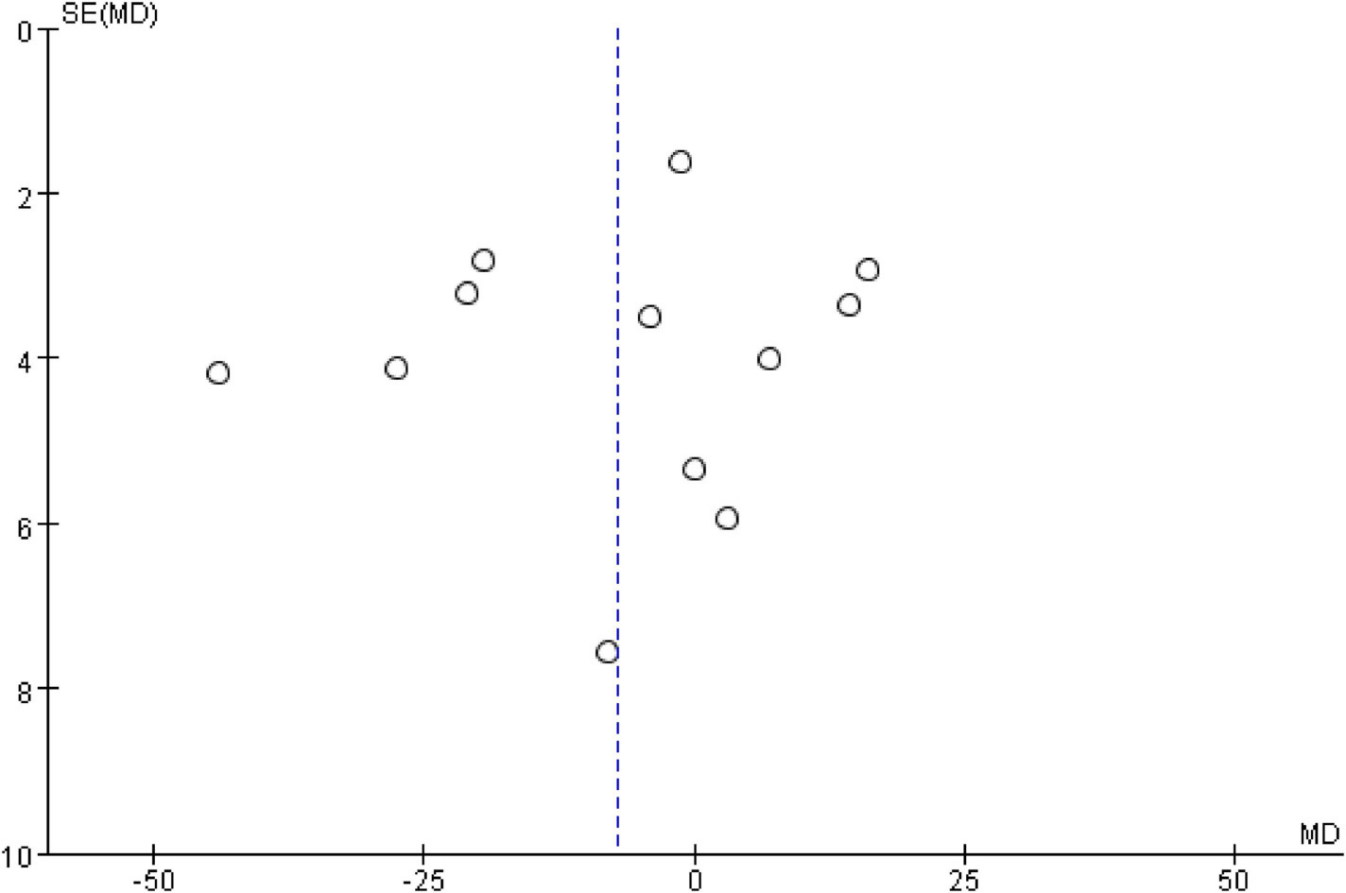
Funnel plot of surgical time.

**Figure 10:**
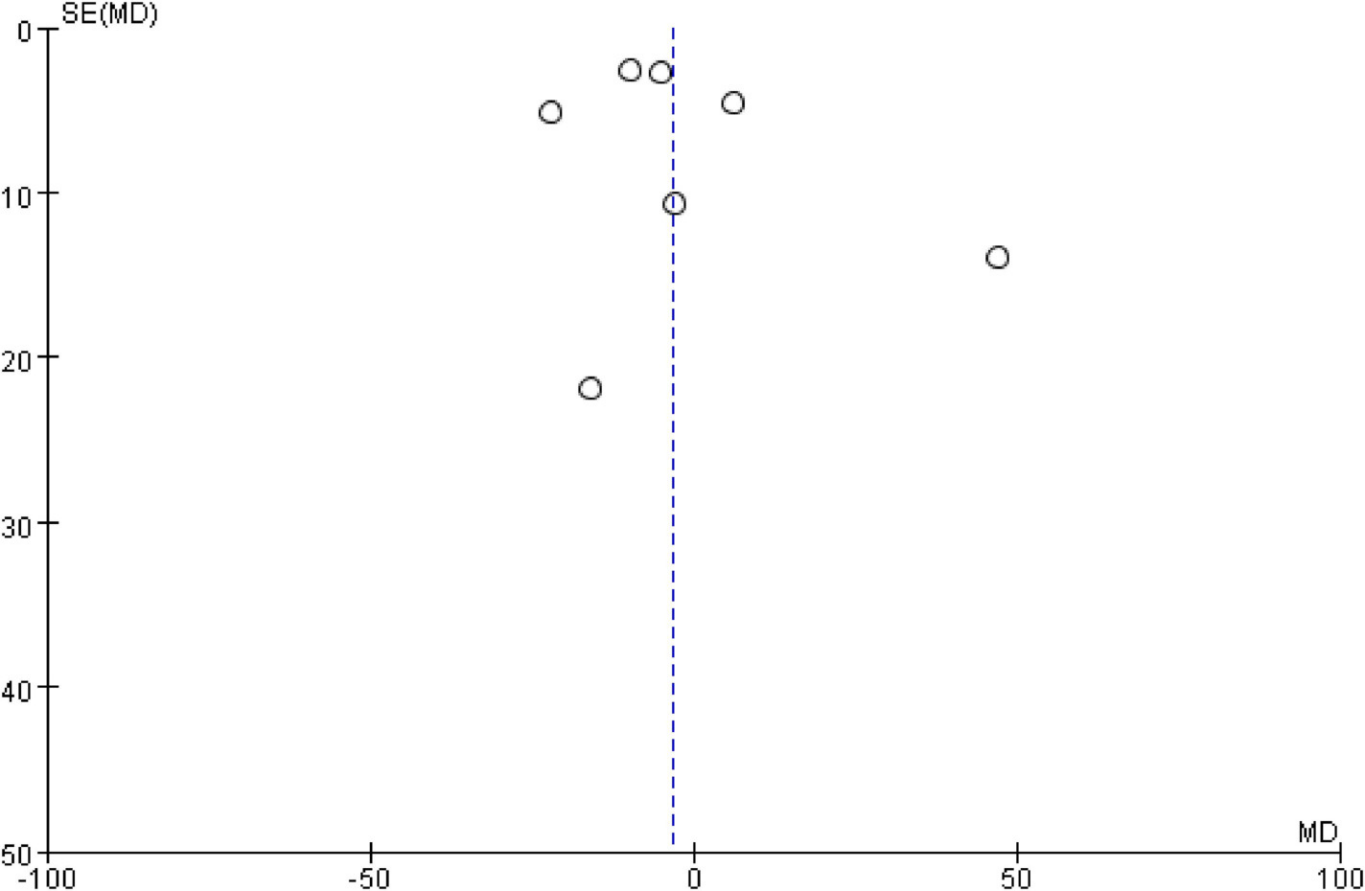
Dose funnel diagram for contrast agent use.

**Figure 11:**
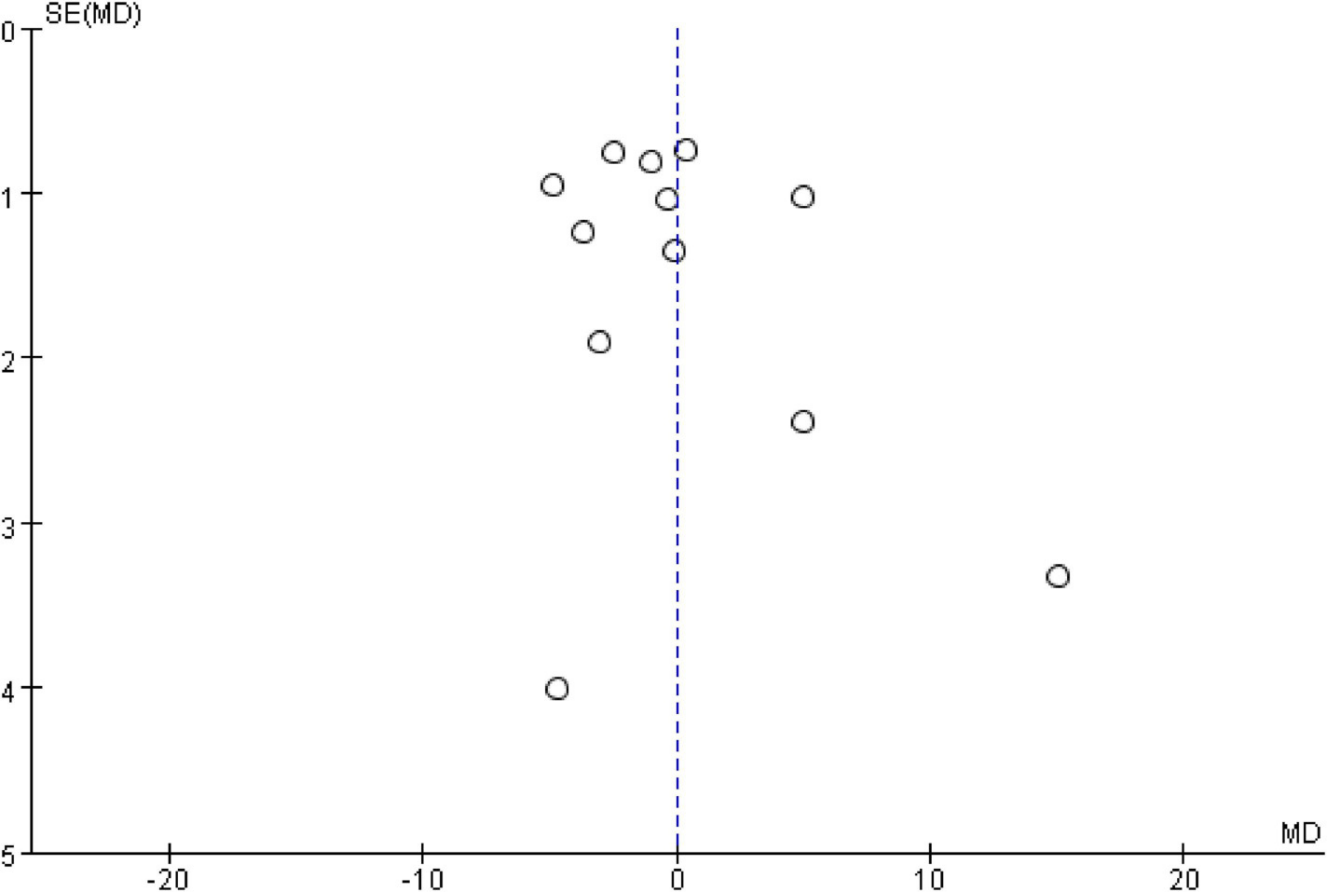
Fluoroscopy times funnel plot.

The inverted funnel plots of the primary and secondary outcomes as independent variables showed that most of the studies on technical success and complication were in the upper part of the “inverted funnel” and fewer were at the base, and the symmetry was roughly left and right, suggesting that there was no obvious publication bias; the data on procedure time, contrast media use, and dominance time of secondary outcomes were in the middle part of the “inverted funnel”, and some studies were at the base, and the symmetry was not obvious, suggesting that there might be a publication bias (See Figures 7, 8). The data on procedure time, contrast use and dominance time for secondary outcomes were in the middle of the “inverted funnel”, and some studies were at the bottom of the “inverted funnel”, and the symmetry was not obvious, suggesting that there might be publication bias (See Figures 9–11). The Egger test did not reach statistical significance (*P* > 0.05), indicating a lack of strong evidence of publication bias.

### 3.7 Assess the quality of the evidence

Based on an assessment of the quality of evidence for image-guided approaches in left atrial appendage occlusion (LAAO), current studies indicate that: although intracardiac echocardiography (ICE) and transesophageal echocardiography (TEE) demonstrate equivalence in core clinical outcomes—technical success rate (RR = 1.01, 95% CI 1.00–1.02) and overall complication risk (RR = 0.94, 95% CI 0.82–1.09), supporting moderate-quality evidence. However, these results are downgraded due to methodological limitations: Technical success rate is constrained by a confidence interval close to the non-effectiveness line (−1 imprecision) and the inclusion of some high-risk studies (−1 bias risk), while the risk of complications requires cautious interpretation due to a wide confidence interval crossing the non-effectiveness line (−1 imprecision) and the dominance of observational designs. Evidence quality for operational efficiency indicators was significantly downgraded to low/very low: Surgical time (MD = −7.12 min, I^2^ = 96%) was highly uncertain due to extreme heterogeneity (−2), conflicting subgroup results (e.g., shorter time in patients under 75 years old but longer time in those with a high proportion of paroxysmal atrial fibrillation, −1 inconsistency), and subjective classification (−1 indirectness); Fluoroscopy time (MD = −0.03 min, I^2^ = 88%) and contrast agent dose (MD = −3.21 mL, I^2^ = 82%) also cannot form reliable recommendations due to residual heterogeneity, device dependency (e.g., multi-seal devices shorten time but carry a risk of selection bias), and unclear clinical significance (wide confidence intervals). Economic indicators (only 3 studies) further highlight contradictions: while ICE reduces professional fees by $2,654 (*P* < 0.001), it increases hospitalization costs by 17.8%, and lacks Chinese data to support universal conclusions, reflecting the limitations of extremely low-quality evidence (Table 6).

**Table 6:** GRADE quality evidence assessment form.

Outcome indicators	Included in study design	Initial mass	Downgrading factors	Upgrading factors	Outcome indicators
Technical success rate	13 observational studies	Low	−1 (risk of bias: includes studies with high risk of bias) −1 (imprecision: RR = 1.01, CI 1.00–1.02, close to the line of no effect)	+1 (strong association: no differences among all subgroups)	Moderate
Overall complication risk	16 observational studies	Low	−1 (Risk of bias: 3 studies with high risk of bias) −1 (Precision: RR = 0.94, CI 0.82–1.09)	+1 (dose effect: consistent trends among all complication subgroups)	Moderate
Surgery time	12 observational studies	Low	−2 (Very high heterogeneity: I^2^ = 96%) −1 (Inconsistency: Subgroup results show conflicting directions) −1 (Indirectness: Subgroup analysis relies on subjective classification)	None	Very low
Contrast agent dose	7 observational studies	Low	−2 (High heterogeneity: I^2^ = 82%) −1 (Precision: MD = −3.21, CI −12.51 to 6.09, crossing zero) −1 (Publication bias: Asymmetric funnel plot)	None	Very low
Fluoroscopy time	12 observational studies	Low	−2 (Extremely high heterogeneity: I^2^ = 88%) −1 (Imprecision: MD = −0.03, CI −2.02 to 1.96) −1 (Indirectness: Subgroup classifications not standardized)	None	Very low
Economic indicators	3 observational studies	Low	−2 (Severe imprecision: Only 3 studies) −1 (Inconsistency: Conflicting results) −1 (Indirectness: Regional variations in cost structures)	None	Very low
Technical success rate	13 observational studies	Low	−1 (risk of bias: includes studies with high risk of bias) −1 (imprecision: RR = 1.01, CI 1.00–1.02, close to the line of no effect)	+1 (strong association: no differences among all subgroups)	Moderate
Overall complication risk	16 observational studies	Low	−1 (Risk of bias: 3 studies with high risk of bias) −1 (Precision: RR = 0.94, CI 0.82–1.09)	+1 (dose effect: consistent trends among all complication subgroups)	Moderate

### 3.8 Clinical interpretation summary based on GRADE

To guide clinical interpretation, findings from this meta-analysis can be stratified by the confidence level derived from the GRADE assessment:

High-Confidence Conclusions (Moderate-Quality Evidence): ICE and TEE are equivalent in technical success rate and overall risk of complications for LAAO. This provides fundamental assurance for clinical choice, supporting ICE as a reliable alternative to avoid general anesthesia, especially in high-risk patients.

Low-Confidence but Suggestive Findings (Very Low-Quality Evidence): Differences in procedural efficiency (operative time, fluoroscopy time, contrast dose) appear moderated by factors such as patient age, AF type, hypertension burden, and device design. For instance, subgroup analyses suggest that ICE may offer advantages in reducing operative time and contrast use in patients aged <75 years, with a low burden of hypertension, and non-paroxysmal AF, particularly when using multi-seal devices. However, due to the very low quality of evidence, these findings are insufficient to form strong recommendations but can serve as considerations for individualized decision-making when faced with specific patient profiles and should be viewed as hypotheses generating for future prospective research.

## 4 Discussions

This systematic review and meta-analysis compared the safety, efficacy, and economics of intracardiac echocardiography (ICE) vs. transesophageal echocardiography (TEE) for guiding left atrial appendage occlusion (LAAO). While several previous meta-analyses have compared ICE and TEE (5, 7, 11, 12, 28), the present study provides updated evidence and deeper insights in the following aspects: First, Data Timeliness: Our search was updated to February 2025, incorporating recent prospective cohort studies published in the last five years (e.g., Qian 2024, Grazina 2023). Second, Analytical Depth: We conducted the most comprehensive pre-specified subgroup analyses to date, systematically exploring for the first time the moderating effects of patient characteristics (age, hypertension, AF type), device design (single-seal vs. multi-seal), and catheter type on outcomes, moving beyond simply reporting inter-group differences. Third, Perspective Expansion: This is the first meta-analysis of its kind to integrate health economic indicators, revealing regional discrepancies and providing initial directions for cost-effectiveness evaluations. Fourth, Systematic Evaluation: We rigorously applied the GRADE framework to rate the quality of evidence, clearly distinguishing robust conclusions (moderate quality for success and safety) from findings requiring cautious interpretation (very low quality for procedural efficiency), thereby offering a clear evidence map for clinical decision-making and future research prioritization.

The results confirm the equivalence of ICE and TEE in terms of technical success (*P* = 0.24) and overall complication risk (*P* = 0.43), reinforcing ICE as a viable alternative to TEE, particularly for patients at high risk for general anesthesia. However, it is noteworthy that a recent large-scale meta-analysis by Serpa et al. (including 19 observational studies and 42,474 patients) reported a higher incidence of pericardial effusion with ICE guidance, despite an overall higher procedural success rate (29). This finding adds nuance to the safety profile comparison, underscoring that the choice between ICE and TEE should not only consider procedural efficiency and operator preference but also weigh potential differences in specific safety outcomes, particularly pericardial complications.However, the in-depth analysis of secondary outcomes revealed significant heterogeneity, and subgroup analyses identified potential key factors influencing procedural efficiency (e.g., operative time, contrast dose), providing evidence-based hypotheses for individualized clinical decision-making.

ICE significantly reduced time in single-center studies, whereas the difference was not significant in multicenter studies, suggesting that insufficient standardization of techniques between centers may diminish the advantages of ICE; ICE significantly reduced operative time when using specific catheters (e.g., AcuNav) and devices with a multiseal design, and the significant reduction in time by ICE may be related to the accumulation of experience by the operator and optimization of the technical process; ICE significantly shortened operative time in patients <75 years old, while the difference was not statistically significant in patients ≥75 years old. patients, ICE significantly shortened the procedure time, while the difference was not statistically significant in patients ≥75 years old, which may be related to more regular LV anatomy and lower operating difficulty in younger patients; while in older patients, the visual field advantage of ICE was weakened by the increased tissue fragility or complex LV morphology (e.g., lobulation, calcification). There was no significant difference in operative time between the ICE group and the TEE group at a proportion of hypertension ≥90%, whereas ICE was significantly shorter at a proportion of hypertension <90%, which may be associated with higher vascular sclerosis and complexity of the left auricular anatomy in hypertensive patients, leading to an increased need for imaging adjustments and weakening of the efficiency advantage of ICE. In the subgroup with a proportion of paroxysmal AF ≥50%, ICE significantly shortened the procedure time (MD = −13.07), whereas it instead increased the procedure time (MD = 14.20) in <50%, and patients with paroxysmal AF were at a lower risk of LV thrombosis, which may reduce the number of intraoperative blocker adjustments and thus enhance the efficiency of ICE operations.

In response to the use of contrast agent usage, ICE significantly reduced the dose in the single-center subgroup, but the dose was inversely increased in the multicenter study, and the use of multi-seal devices also reflected a reduction in dose, which may be related to differences in the operating habits of ICE catheters (e.g., repeatedly adjusting the position of the catheter) between different centers, suggesting a bi-directional effect of the operating environment and the choice of instrumentation on the dose; the age of <75 years and the male ICE significantly reduced contrast dosage when the proportion was ≥70%, possibly due to better vascularization in younger male patients, which resulted in better definition of ICE imaging and reduced reliance on contrast.

When subgroups of fluoroscopic time were analyzed, ICE significantly shortened duration in the sample size ≤100 and multiple-seal device subgroups (*P* < 0.05), but TEE was superior with the single-seal device, reflecting the potential modulation of imaging efficiency by device design. ICE significantly shortened fluoroscopic time with the AcuNav catheter in patients <75 years of age, whereas the ViewFlex catheter did not demonstrate an advantage in patients ≥75 years of age. This may be due to the greater flexibility of the AcuNav catheter, which is better suited to the anatomical characteristics of younger patients, whereas older patients require more fluoroscopy to confirm the location of the occluder. There was no difference between ICE and TEE fluoroscopic time in the subgroup with ≥90% hypertension, whereas ICE was significantly shorter in <90%. Hypertensive patients may require more frequent fluoroscopic confirmation because of irregular LV morphology (e.g., multilobar, calcification), counteracting the local imaging advantage of ICE.

As a core intervenable risk factor for cardiovascular disease, hypertension can exacerbate AF progression and stroke risk by promoting left atrial dilatation, atrial fibrosis, and endothelial dysfunction (7). In this study, we found that in the subgroup with a prevalence of hypertension <90%, ICE guidance significantly shortened the procedure time, reduced contrast dosage, and fluoroscopic time compared with TEE, suggesting that patients with a low hypertension burden are more likely to benefit from ICE guidance. This phenomenon may be related to hypertension-associated vascular sclerosis and LV morphologic complexity (e.g., multifractionation, calcification), the latter of which may increase the frequency of ICE catheter imaging adjustments, thereby diminishing its efficiency advantage. This finding provides a key entry point for subsequent studies on the relationship between hypertension severity and choice of imaging guidance modality.

The type of AF significantly affects the efficiency of LAAO operation, and according to the German LAARGE registry study, patients with paroxysmal AF (PAF) had 21% longer operation time and fluoroscopic time compared with non-PAF patients due to the higher percentage of intraoperative sinus rhythm, which resulted in increased left auricular motility (30). The present subgroup analysis further revealed that when the percentage of PAF was ≥50%, the procedure time in the ICE group was prolonged by 14.2 min compared with the TEE group, while the contrast dosage in the ICE group was reduced by 38.2 mL when PAF was <50%.This difference may stem from two factors: (i) increased LV systolic activity in sinus rhythm, which required more frequent ICE image calibration; and (ii) risk of LV thrombosis was higher in patients with non-PAF, prompting the need for ICE image calibration in the TEE group The higher risk of left auricular thrombosis in non-PAF patients prompted the need for more contrast in the TEE group to validate the effectiveness of blockade.

Existing LAAO devices can be categorized by sealing mechanism into single-seal (e.g., Amplatzer Cardiac Plug), double-seal (e.g., WATCHMAN FLX), and hybrid (30). Although clinical outcomes are similar between the different types of devices (31), this study found that multiseal devices demonstrated significant advantages under ICE guidance: shorter procedure times and lower contrast dosage. This may be due to the synergistic effect of the dual fixation mechanism of the multiseal device and the real-time 3D imaging of ICE, but we need to be wary of selection bias - operators tend to use ICE in combination with advanced sealing devices for complex anatomical cases. Future prospective studies are needed to clarify the optimal device-imaging matching strategy.

ICE reduces professional costs (e.g., anesthesia costs, MD = −2654 USD, *P* < 0.001), but its catheter costs and hospitalization costs (e.g., 17.8% higher hospitalization costs in the ICE group in Morcos 2022) lead to conflicting overall economics, suggesting that the choice needs to be weighed in relation to specific healthcare resource allocation strategies. Based on the cost data analysis in the United States, the total costs of ICE and TEE are comparable or even lower, but in China, since the cost of general anesthesia for TEE is much lower than the cost of catheterization for ICE, the total cost of ICE-guided LAAO may be higher than that of TEE in clinical practice, but there is a lack of comparative economic research on its application within China. Future studies should conduct a cost-utility analysis of ICE and TEE based on cost comparisons, fully consider patients’ satisfaction with their improved health status, and select a more economical intraoperative imaging modality for LAAO.

Although there have been five previous Meta-analyses (5, 7, 11, 12, 28) focusing on the safety and efficacy of ICE vs. TEE-guided LAAO, the latest search deadline for them was 2022, and several relevant new prospective cohort studies have been published in the last 5 years. The present study was supplemented with the inclusion of the most recent studies, with full attention to all important outcome indicators, separate quantitative analyses of multiple major complications, more comprehensive and objective findings, and the first focus on economics-related indicators. In addition, sensitivity analyses and subgroup analyses were conducted to assess the stability of the study results. All previous Meta-analyses showed no difference between ICE and TEE-guided LAAO intraoperative imaging in terms of procedural success rate, procedure-related complications, procedure time, and fluoroscopy time, which is consistent with the results of this study. Regarding contrast dose, the Meta-analysis by Ribeiro et al. (28), which included 2 studies, showed that the ICE group used less contrast dose than the TEE group, whereas the combined results of Jhand et al. (5), which included 5 studies, and of the present study, which included 8 studies, showed that there was no statistically significant difference in contrast dose between the two groups.

Inter-study heterogeneity was particularly prominent in the secondary outcomes, mainly due to the following factors: (1) study design and sample size: single-center studies were more likely to demonstrate the efficiency advantage of ICE due to the centralized procedure and uniform operator experience (e.g., operation time I^2^ = 96%), while multicenter studies included teams with different levels of experience, which led to dispersed results (e.g., multicenter MD = 47.00 vs. single-center MD = −7.15 in the subgroup of contrast dose); (2) device and catheter type: blocker design (single-seal vs. double-seal) directly affected the complexity of operation due to structural characteristics. device and catheter type: the design of the occluder (single-seal vs. dual-seal) directly affects the complexity of the operation, and dual-seal devices (e.g., Amulet) may require more precise imaging due to structural characteristics, which can magnify the field of view limitations of the ICE (e.g., MD of time to surgery in the multi-seal device subgroup = −19.75); catheter type also significantly affects the results, with the ViewFlex catheter having a significant impact on the results. ViewFlex catheters had better TEE in the procedure time subgroup (MD = 20.80), whereas the opposite was true for AcuNav catheters (MD = −3.21), possibly related to differences in the imaging field of view and catheter maneuverability; (iii) Patient characteristics: age stratification (<75 vs. ≥75 years) and type of AF (paroxysmal predominance) indirectly influenced the results through anatomical and hemodynamic variations. (iii) Patient characteristics: age stratification (<75 years vs. ≥75 years) and type of AF (paroxysmal ratio) indirectly influence the difficulty of manipulation through anatomic and hemodynamic changes. For example, more regular LV morphology in patients <75 years of age may reduce the field of view limitations of ICE (fluoroscopic time MD = −1.98), whereas older patients (≥75 years of age) may offset the technical advantages due to increased tissue fragility; (iv) economic indicators of ambivalence: although ICE reduces professional costs (MD = −$2,654), it is associated with an increase in catheterization costs and hospitalization costs (e.g., hospitalization costs were 17.8% higher in the ICE group in Morcos 2022), the overall economics need to be assessed in combination with the allocation of healthcare resources.

Our meta-analysis revealed substantial heterogeneity (I^2^ > 90%) for several secondary outcomes, including procedural time, contrast volume, and fluoroscopy time, indicating considerable variation in effect sizes across the included studies. Despite our subgroup analyses identifying some potential sources (e.g., study design, device type, patient age), significant residual heterogeneity likely reflects unmeasured confounders. A key factor is operator experience and the learning curve associated with ICE-guided LAAO. Proficiency in ICE catheter manipulation and interpretation varies considerably between operators and centers, directly impacting procedural efficiency metrics like time and contrast use. Studies did not consistently report operator experience levels, limiting our ability to adjust for this factor. Center volume and procedural standardization are also critical. High-volume centers with established ICE protocols likely achieve greater efficiency gains compared to centers with lower volumes or less standardized approaches. The multicenter studies, inherently incorporating this variability, showed less pronounced benefits or even disadvantages for ICE in some analyses (e.g., contrast volume), underscoring the impact of center-specific factors. Furthermore, rapid evolution in ICE catheter technology and LAAO device design occurred during the study period. Earlier studies might have used less advanced ICE systems or older device generations, potentially contributing to the observed heterogeneity. Our analyses attempted to account for device type (seal mechanism) and specific ICE catheters where possible, but granular data on specific device iterations or ICE system capabilities were often lacking. This high heterogeneity necessitates caution in interpreting the pooled estimates for these secondary outcomes. While the subgroup analyses provide valuable insights into potential modifiers of the ICE vs. TEE effect, the point estimates should be viewed as representing an average effect across diverse settings rather than a precise, universally applicable value. The direction of effect in specific subgroups (e.g., benefit of ICE in younger patients, with multi-seal devices) remains informative for hypothesis generation and clinical decision-making considering local context.

Based on GRADE, ICE and TEE are equivalent in terms of technical success rate and safety (moderate-quality evidence), but the evidence quality regarding differences in procedural efficiency is extremely low (severely limited by heterogeneity and bias). ICE is recommended for young patients (<75 years old) with non-paroxysmal atrial fibrillation and simple anatomy (weak recommendation), while also considering team experience and health economic assessments (e.g., ICE should be prioritized for patients with high anesthesia risks). High-quality RCTs are needed in the future to validate operational efficiency and cost-effectiveness.

The limitations of this study must be carefully considered. First, the included studies were exclusively observational in design (8 retrospective and 8 prospective cohorts), with no randomized controlled trials (RCTs). This inherent limitation prevents full control for confounding biases. Although we used the ROBINS-I tool for assessment, three studies (Reis 2018, Su 2022, Du Xianfeng 2021) were rated as having a high risk of bias, which may affect the accuracy of subgroup and economic analyses derived from the pooled data.Second, we observed extreme statistical heterogeneity (I^2^ > 90%) for key secondary outcomes (operative time, fluoroscopy time, contrast dose). Although we explored sources through extensive subgroup analyses (e.g., study center, age, device type), significant residual heterogeneity suggests the presence of important unmeasured confounders. These likely include: (1) Operator experience and learning curve: Proficiency in ICE manipulation varies significantly among operators and centers, directly impacting procedural efficiency metrics, a factor inconsistently reported in primary studies; (2) Center volume and procedural standardization: High-volume centers with standardized protocols may better realize ICE's efficiency advantages, whereas multicenter studies amalgamate experiences from centers of varying proficiency, potentially diluting these advantages; (3) Rapid technological evolution: Iterative advancements in ICE catheter technology and LAAO device design during the study period may affect the comparability of results between earlier and later studies. Consequently, the pooled estimates for procedural efficiency metrics should be interpreted as average effects across diverse clinical settings rather than precise, universally applicable values. Third, the economic analysis was based on only three studies with conflicting findings (e.g., ICE reduced professional fees but potentially increased hospitalization costs). Crucially, the lack of region-specific cost data, particularly from key markets like China, severely limits the generalizability and practical application of our economic conclusions. This underscores the necessity for future cost-utility analyses based on local healthcare pricing structures. Finally, the follow-up duration across included studies was relatively short (1–12 months), preventing assessment of the impact of either imaging modality on long-term outcomes (e.g., device-related thrombosis, late stroke). Future studies with longer follow-up are needed to comprehensively evaluate long-term benefits and risks. Despite these limitations, this study offers a timely synthesis of current evidence, delivers novel insights through comprehensive subgroup and economic analyses, and provides a transparent evaluation of evidence quality using the GRADE framework, guiding both clinical practice and future research.

It is important to emphasize that robust randomized controlled trials (RCTs) directly comparing ICE and TEE for LAAO guidance are currently lacking. This evidence gap limits the strength of recommendations derived from observational data. The recently initiated ICE-TEE trial (Al-Azizi et al.) represents a pivotal step forward (32, 33). This prospective, randomized study is expected to provide high-quality evidence that may clarify the comparative efficacy, safety, and potential advantages of each imaging modality, ultimately informing future clinical guidelines and practice standards.Additionally, anesthesia-related complications were not systematically reported across the included studies. Furthermore, it was often not specified whether procedures in the TEE group were uniformly performed under general anesthesia or whether conscious sedation was employed in some cases. This lack of detailed peri-procedural management data limits a nuanced comparison of the safety profiles between the two strategies, particularly regarding anesthesia-related adverse events.

## 5 Reach a verdict

ICE and TEE have equivalent core clinical values in LAAO, but their operational efficiency and economy are regulated by multiple factors, including patient age, instrument design, and operator experience. Clinical decisions need to be individually weighed, and ICE is preferentially recommended for young, anatomically simple patients, and optimized for technical suitability in combination with team experience.

## Data Availability

The original contributions presented in the study are included in the article/Supplementary Material, further inquiries can be directed to the corresponding author.
