# Supplementary material for: Systematic evaluation and meta-analysis of transcardiac intracavitary and transesophageal echocardiography-guided left atrial appendage occlusion surgery
Source: Front Cardiovasc Med. 2026 Mar 3;13:1701359. doi: 10.3389/fcvm.2026.1701359 (PMC12992318; doi:10.3389/fcvm.2026.1701359)
Supplement: Supplementary file 1 [file Supplementaryfile1.docx]

1. **PICU Resident #1**
   1. Ask family to join rounds
   2. 1-line patient introduction
   3. Overnight events
2. **Nurse**
   1. Presents from the nurse rounding script
3. **PICU Resident #1**
   1. Physical exam findings
   2. Labs
   3. Images
   4. Overall assessment
   5. System-based plan
      1. Organ systems can be presented in order of resident preference
      2. RT, pharmacist, dietician, and nurse may chime in for data corrections
4. **RT**
   1. Unaddressed issues 🡪 resident to address issues
5. **Pharmacist**
   1. Unaddressed issues 🡪 resident to address issues
   2. If any pertinent meds were not discussed, can bring them up; if the consultant requests for a med list, please read off
6. **Dietitian**
   1. Unaddressed issues 🡪 resident to address issues
7. **Nurse**
   1. Any final unaddressed issues 🡪 resident to address issues
8. **PICU Resident #2**
   1. Complete PICU Daily Rounding Checklist by non-presenting resident
      1. Checklist can be initiated while Resident #1 presents plan; if a topic was discussed, check off the “Was Already Addressed or N/A” box
      2. Only need to verbalize tasks not addressed in rounds up to this point
         1. If the final plan changed because of the checklist, check off the “Checklist Helped Change Plan” box
         2. If the final plan did not change despite verbalizing topics not addressed in rounds up to this point, check off the “Was Already Addressed or N/A” box
9. **PICU Consultant**
   1. Update and summarize the final plan
   2. Ask family for additional questions/concerns 🡪 **PICU Resident #1**/PICU Consultant to address issues
10. **PICU Resident #3**
    1. Should be placing orders during pt presentation
    2. Order read back by non-presenting, non-checklist completing resident
