# Supplementary material for: Systematic evaluation and meta-analysis of transcardiac intracavitary and transesophageal echocardiography-guided left atrial appendage occlusion surgery
Source: Front Cardiovasc Med. 2026 Mar 3;13:1701359. doi: 10.3389/fcvm.2026.1701359 (PMC12992318; doi:10.3389/fcvm.2026.1701359)
Supplement: Supplementary file 2 [file Supplementaryfile2.docx]

Subgroup analysis table of surgical time

| Subgroup factors | Numbers of study | RR (95%CI) | I^2^ (%) | *P* value | *P* for interaction |
| --- | --- | --- | --- | --- | --- |
| Study design |  |  |  |  | 0.37 |
| Single-center | 8 | 1.00(0.98, 1.02) | 0 | 0.98 |  |
| Multi-center | 5 | 1.01(0.99, 1.03) | 0 | 0.21 |  |
| ICE Sample size |  |  |  |  | 0.87 |
| ≤100 | 8 | 1.00(0.99, 1.02) | 0 | 0.60 |  |
| >100 | 5 | 1.01(0.99, 1.02) | 0 | 0.37 |  |
| Male proportion |  |  |  |  | 0.25 |
| <70 | 9 | 1.01(0.99, 1.02) | 0 | 0.22 |  |
| ≥70 | 4 | 1.00(0.98, 1.01) | 0 | 0.63 |  |
| Age cutoff |  |  |  |  | 0.80 |
| <75 | 7 | 1.00(0.99, 1.02) | 0 | 0.62 |  |
| ≥75 | 6 | 1.00(0.99, 1.02) | 0 | 0.37 |  |
| HT proportion |  |  |  |  | 0.74 |
| <90 | 7 | 1.00(0.98, 1.02) | 0 | 0.88 |  |
| ≥90 | 2 | 1.00(0.97, 1.02) | 0 | 0.77 |  |
| PAF proportion |  |  |  |  | 0.63 |
| ≤50 | 5 | 1.01(0.99, 1.04) | 11 | 0.20 |  |
| >50 | 1 | 1.00(0.95, 1.05) | - | 1 |  |
| Devices type |  |  |  |  | 0.75 |
| Dual-seal mechanism | 5 | 1.00(0.98, 1.02) | 0 | 0.87 |  |
| Single-seal mechanism | 4 | 1.01(0.99, 1.03) | 0 | 0.25 |  |
| Muti-seal mechanism | 4 | 1.01(0.99, 1.02) | 0 | 0.82 |  |
| ICE Catheter Type |  |  |  |  | 0.44 |
| AcuNav | 4 | 1.02(0.99, 1.05) | 0 | 0.19 |  |
| ViewFlex | 3 | 1.00(0.99, 1.01) | 0 | 0.84 |  |
| integrated | 2 | 1.01(0.97, 1.03) | 0 | 0.87 |  |

Note: ICE: intracardiac echocardiography; TEE: transesophageal echocardiography; RR:relative risk; CI: confidence interval.
