# Supplementary material for: Systematic evaluation and meta-analysis of transcardiac intracavitary and transesophageal echocardiography-guided left atrial appendage occlusion surgery
Source: Front Cardiovasc Med. 2026 Mar 3;13:1701359. doi: 10.3389/fcvm.2026.1701359 (PMC12992318; doi:10.3389/fcvm.2026.1701359)
Supplement: Supplementary file 3 [file Supplementaryfile3.docx]

Table for subgroup analysis of doses of contrast media used

| Subgroup factors | Numbers of study | MD (95%CI) | I^2^ (%) | *P* value | *P* for interaction |
| --- | --- | --- | --- | --- | --- |
| Study design |  |  |  |  | 0.0008 |
| Single-center | 8 | -7.28(-9.46, -5.10) | 96 | ＜0.00001 |  |
| Multi-center | 4 | -0.40(-3.76, 2.97) | 96 | 0.82 |  |
| ICE Sample size |  |  |  |  | 0.002 |
| ≤100 | 6 | -3.02(-5.33, -0.72) | 92 | 0.01 |  |
| >100 | 6 | -9.07(-12.09, -6.04) | 97 | ＜0.00001 |  |
| Male proportion |  |  |  |  | 0.0002 |
| <70 | 8 | -3.42(-5.49, -1.36) | 94 | 0.0001 |  |
| ≥70 | 4 | 0.67(-1.61, 2.96) | 98 | ＜0.00001 |  |
| Age cutoff |  |  |  |  | ＜0.00001 |
| <75 | 6 | -15.89(-18.95, -12.82) | 86 | ＜0.00001 |  |
| ≥75 | 6 | -12.11(-16.12, -8.11) | 97 | 0.56 |  |
| HT proportion |  |  |  |  | 0.02 |
| <90 | 5 | -12.00(-15.08, -8.92) | 96 | ＜0.00001 |  |
| ≥90 | 2 | -7.05(-10.03, -4.07) | 99 | ＜0.00001 |  |
| PAF proportion |  |  |  |  | ＜0.00001 |
| ≤50 | 7 | -13.07(-15.71, -10.44) | 11 | ＜0.00001 |  |
| >50 | 1 | 14.20(7.60, 20.80) | - | ＜0.00001 |  |
| Devices type |  |  |  |  | ＜0.00001 |
| Dual-seal mechanism | 4 | 1.54(-1.16, 4.23) | 83 | 0.26 |  |
| Single-seal mechanism | 3 | 2.85(1.18, 6.87) | 97 | 0.17 |  |
| Muti-seal mechanism | 5 | -19.75(-22.93, -16.56) | 93 | ＜0.00001 |  |
| ICE Catheter Type |  |  |  |  | ＜0.00001 |
| AcuNav | 4 | -3.21(-6.20, -0.19) | 98 | 0.04 |  |
| ViewFlex | 3 | 20.80(25.44, -16.16) | 98 | ＜0.00001 |  |
| integrated | 2 | -1.28(-4.34, 1.78) | 0 | 0.41 |  |
| SoundStar | 1 | -8.10(-22.80, 6.70) | - | 0.28 |  |

Note: ICE: intracardiac echocardiography; TEE: transesophageal echocardiography; MD: mean difference; CI: confidence interval.
