# Supplementary material for: Systematic evaluation and meta-analysis of transcardiac intracavitary and transesophageal echocardiography-guided left atrial appendage occlusion surgery
Source: Front Cardiovasc Med. 2026 Mar 3;13:1701359. doi: 10.3389/fcvm.2026.1701359 (PMC12992318; doi:10.3389/fcvm.2026.1701359)
Supplement: Supplementary file 4 [file Supplementaryfile4.docx]

Analysis table of perspective hours subgroups

| Subgroup factors | Numbers of study | MD (95%CI) | I^2^ (%) | *P* value | *P* for interaction |
| --- | --- | --- | --- | --- | --- |
| Study design |  |  |  |  | 0.0001 |
| Single-center | 6 | -7.15(-10.33, -3.96) | 73 | ＜0.00001 |  |
| Multi-center | 1 | 47.00(19.58, 74.42) | - | 0.0008 |  |
| ICE Sample size |  |  |  |  | 0.50 |
| ≤100 | 3 | -5.03(-10.13, 0.07) | 0 | 0.05 |  |
| >100 | 4 | -7.29(-11.32, -3.27) | 91 | 0.0004 |  |
| Male proportion |  |  |  |  | 0.0009 |
| <70 | 4 | -1.03(-5.51, 3.36) | 82 | 0.65 |  |
| ≥70 | 3 | -11.76(-16.21,-7.30) | 63 | ＜0.00001 |  |
| Age cutoff |  |  |  |  | 0.43 |
| <75 | 2 | -9.69(-14.77, -4.61) | 0 | 0.0002 |  |
| ≥75 | 4 | -6.95(-11.45, -2.44) | 88 | 0.003 |  |
| HT proportion |  |  |  |  | 0.86 |
| <90 | 3 | -9.32(-14.26, -4.37) | 0 | 0.0002 |  |
| ≥90 | 2 | -8.72(-13.40, -4.04) | 88 | 0.0003 |  |
| PAF proportion |  |  |  |  | 0.40 |
| ≤50 | 2 | -12.17(-16.73, -7.61) | 79 | ＜0.00001 |  |
| >50 | 1 | -2.90(-23.79, 18.17) | - | 0.79 |  |
| Devices type |  |  |  |  | 0.001 |
| Dual-seal mechanism | 3 | -2.22(-6.69, 2.24) | 53 | 0.33 |  |
| Single-seal mechanism | 2 | -7.69(-12.73, -2.66) | 94 | 0.003 |  |
| Muti-seal mechanism | 2 | -21.69(-31.44, -11.94) | 0 | ＜0.0001 |  |
| ICE Catheter Type |  |  |  |  | 0.26 |
| AcuNav | 1 | -2.90(-23.97, -18.17) | - | 0.79 |  |
| ViewFlex | 3 | -10.58(-15.08, -6.08) | 98 | ＜0.00001 |  |
| integrated | 1 | -5.00(-10.30,0.30) | - | 0.06 |  |

Note: ICE: intracardiac echocardiography; TEE: transesophageal echocardiography; MD: mean difference; CI: confidence interval.
