# Supplementary material for: Systematic evaluation and meta-analysis of transcardiac intracavitary and transesophageal echocardiography-guided left atrial appendage occlusion surgery
Source: Front Cardiovasc Med. 2026 Mar 3;13:1701359. doi: 10.3389/fcvm.2026.1701359 (PMC12992318; doi:10.3389/fcvm.2026.1701359)
Supplement: Supplementary file 5 [file Supplementaryfile5.docx]

Analysis table of perspective hours subgroups

| Subgroup factors | Numbers of study | MD (95%CI) | I^2^ (%) | *P* value | *P* for interaction |
| --- | --- | --- | --- | --- | --- |
| Study design |  |  |  |  | 0.32 |
| Single-center | 8 | -1.00(-1.78, -0.22) | 85 | 0.01 |  |
| Multi-center | 4 | 0.33(1.38, 0.71) | 94 | 0.53 |  |
| ICE Sample size |  |  |  |  | 0.0002 |
| ≤100 | 7 | -1.72(-2.52, -0.92) | 88 | ＜0.0001 |  |
| >100 | 5 | -0.75(-0.25, -1.76) | 87 | 0.14 |  |
| Male proportion |  |  |  |  | 0.05 |
| <70 | 8 | -0.29(-1.07, 0.49) | 92 | 0.47 |  |
| ≥70 | 4 | -1.61(-2.66,-0.56) | 33 | 0.003 |  |
| Age cutoff |  |  |  |  | 0.0007 |
| <75 | 6 | -1.98(-2.92, -1.03) | 88 | ＜0.0001 |  |
| ≥75 | 6 | -0.19(-0.64, -1.03) | 88 | 0.65 |  |
| HT proportion |  |  |  |  | 0.09 |
| <90 | 5 | -9.32(-14.26, -4.37) | 87 | 0.003 |  |
| ≥90 | 2 | -0.05(-1.40, -1.31) | 64 | 0.95 |  |
| PAF proportion |  |  |  |  | 0.47 |
| ≤50 | 6 | -1.14(-2.13, -0.15) | 91 | 0.02 |  |
| >50 | 1 | -0.10(-2.75, 2.55) | - | 0.94 |  |
| Devices type |  |  |  |  | ＜0.0001 |
| Dual-seal mechanism | 4 | -0.93(-1.75, -0.10) | 62 | 0.03 |  |
| Single-seal mechanism | 3 | 2.56(1.19, 3.94) | 86 | 0.0002 |  |
| Muti-seal mechanism | 5 | -3.46(-4.79, -2.13) | 88 | ＜0.00001 |  |
| ICE Catheter Type |  |  |  |  | ＜0.00001 |
| AcuNav | 3 | -1.06(-0.28, 2.40) | 93 | 0.12 |  |
| ViewFlex | 3 | -0.23(-1.90, 1.45) | 71 | 0.79 |  |
| integrated | 2 | -1.02(-2.06,0.02) | 87 | 0.05 |  |
| SoundStar | 1 | -4.90(-6.78,-3.02) | - | ＜0.00001 |  |

Note: ICE: intracardiac echocardiography; TEE: transesophageal echocardiography; MD: mean difference; CI: confidence interval.
