# Supplementary material for: Systematic evaluation and meta-analysis of transcardiac intracavitary and transesophageal echocardiography-guided left atrial appendage occlusion surgery
Source: Front Cardiovasc Med. 2026 Mar 3;13:1701359. doi: 10.3389/fcvm.2026.1701359 (PMC12992318; doi:10.3389/fcvm.2026.1701359)
Supplement: Supplementary file 6 [file Supplementaryfile6.docx]

1. Misleading Title: “Left Ear Occlusion” should be corrected to “Left Atrial Appendage Occlusion” to reflect clinical terminology.

Thank you for your suggestions; the revisions have been made, and similar errors in the manuscript have also been corrected.

1. The manuscript occasionally suffers from awkward phrasing and grammatical errors (e.g., “left ear blockade” instead of “LAAO”). Repetitive phrasing (e.g., restating objectives in multiple places) could be streamlined.

Thank you for your suggestions. The revisions have been made, and the simplifications have been checked and corrected for grammar.

c) Statistical Part:

1. High heterogeneity (I² > 90% in many outcomes) is downplayed.

While subgroup analyses are conducted, residual heterogeneity suggests underlying confounders remain unaccounted for (e.g., operator experience, center volume, device iterations).

Thank you for your feedback, revisions have been made. The entire subgroup analysis section has been re-described, and the specific results of the subgroup analysis are submitted in the supplementary file.

2. The publication bias analysis relies only on funnel plots; statistical tests (e.g., Egger’s regression) would strengthen conclusions.

No GRADE assessment of evidence quality is included — this would enhance the robustness of clinical recommendations.

Thank you for your suggestions. The revisions have been made, and a GRADE table has been added to the manuscript.

d) Figures (like forest plots and funnel plots) are mentioned but not available for reader evaluation (in this version).

Consider including summary of findings tables for clarity.

Thank you for your suggestions. The modifications have been made, the images reflecting the results have been optimized, and the results have been re-described and summarized.
